# The Robustness of a Signaling Complex to Domain Rearrangements Facilitates Network Evolution

**DOI:** 10.1371/journal.pbio.1002012

**Published:** 2014-12-09

**Authors:** Paloma M. Sato, Kogulan Yoganathan, Jae H. Jung, Sergio G. Peisajovich

**Affiliations:** Department of Cell and Systems Biology, and Centre for the Analysis of Genome Evolution and Function, University of Toronto, Toronto, Ontario, Canada; University of Bath, United Kingdom

## Abstract

The rearrangement of protein domains is known to have key roles in the evolution of signaling networks and, consequently, is a major tool used to synthetically rewire networks. However, natural mutational events leading to the creation of proteins with novel domain combinations, such as *in frame* fusions followed by domain loss, retrotranspositions, or translocations, to name a few, often simultaneously replace pre-existing genes. Thus, while proteins with new domain combinations may establish novel network connections, it is not clear how the concomitant deletions are tolerated. We investigated the mechanisms that enable signaling networks to tolerate domain rearrangement-mediated gene replacements. Using as a model system the yeast mitogen activated protein kinase (MAPK)-mediated mating pathway, we analyzed 92 domain-rearrangement events affecting 11 genes. Our results indicate that, while domain rearrangement events that result in the loss of catalytic activities within the signaling complex are not tolerated, domain rearrangements can drastically alter protein interactions without impairing function. This suggests that signaling complexes can maintain function even when some components are recruited to alternative sites within the complex. Furthermore, we also found that the ability of the complex to tolerate changes in interaction partners does not depend on long disordered linkers that often connect domains. Taken together, our results suggest that some signaling complexes are dynamic ensembles with loose spatial constraints that could be easily re-shaped by evolution and, therefore, are ideal targets for cellular engineering.

## Abstract 2

The broad tolerance of domain-rearranging mutations by a yeast signaling network suggests that signaling complexes have loose spatial constraints, making manipulation and perhaps evolution easier.

## Author Summary

Cells use complex protein interaction networks to sense and process external signals. Proteins involved in signaling are often composed of multiple functional units called domains. Because domains are modular, mutations that rearrange domains among proteins have the potential to result in the creation of novel proteins with altered functions. At an evolutionary timescale, domain rearrangements contribute to the functional diversification of signaling networks; at the shorter timescale of the life of an individual, domain rearrangements can impair cellular functions and lead to disease. Here, we investigated how domain-rearranging mutations alter the function of signaling networks, in particular when these mutations disrupt pre-existing proteins. We used as a model system the yeast mating signaling pathway, which shares many properties with more complex pathways active in human cells. Our results demonstrate that signaling networks are often robust to domain rearrangements that disrupt pre-existing genes. In addition, our experiments suggest a possible mechanism to explain this robustness: rather than being a rigid multi-protein machine, the yeast mating signaling complex is a dynamic ensemble with loose spatial constraints. Because of this, the changes in protein interaction partners caused by domain-rearrangement mutations can be accommodated without disrupting network function.

## Introduction

Cell signaling networks possess a remarkably modular organization. This modularity has attracted the attention of synthetic biologists, for it offers a plausible approach to engineer novel and useful cellular behaviors. At the center of this modular organization are protein domains, which are recurring structural units that often perform modular and thus portable functions [1],[2]. In most signaling proteins, multiple domains are connected by flexible linkers [3]. Diverse genetic mechanisms can rearrange domains, leading to the creation of proteins with novel domain combinations [4]–[8], and thus altered functions [9]–[11]. While, the most prevalent mechanism is gene duplication and in frame fusion, followed by the loss of terminal domains [12], other mechanisms such as transpositions, translocations, inversions, or recombinations, though less prevalent, can also lead to the same functional outcome [4].

Experimental [9],[11] and computational [5],[10],[13]–[15] efforts have revealed that domain rearrangements play a prominent role in the mutational re-wiring of signaling networks, with clear consequences for evolution [4],[12] and disease [16]. Furthermore, the versatility conferred by the functional modularity of protein domains, has begun to be harnessed by protein engineers and synthetic biologists [17]–[19], and promises to open new avenues for cellular engineering [20].

**Figure 1:**
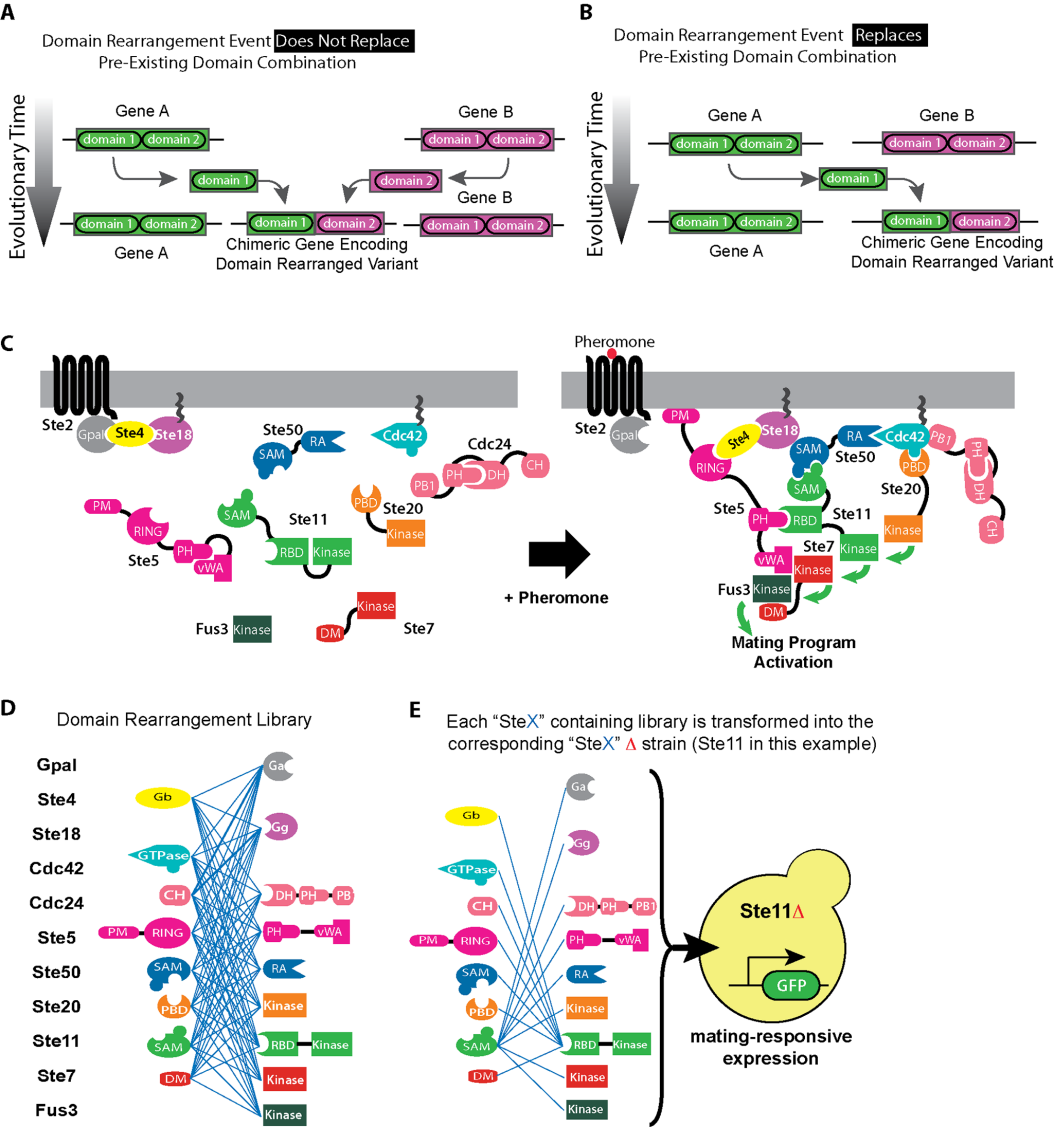
Experimental strategy. (A) Domain-rearrangement event creates a protein with a new domain combination. Still, a previous duplication ensures that at least one copy of the original gene with a pre-existing domain combination is maintained. (B) domain-rearrangement event creates a protein with a new domain combination, while simultaneously replacing a pre-existing gene. (C) The yeast mating pathway is activated by binding of a pheromone to a GPCR (Ste2 in “a” cells and Ste3 in “α” cells), which leads to the dissociation of the Gα subunit from the Gβγ subunits. Subsequent recruitment of the Ste5 scaffold brings three kinases to the membrane proximity (the MAP3K Ste11, the MAP2K Ste7, and the MAPK Fus3). The interaction of the adaptor Ste50 with the small GTPase Cdc42 connects the p21-activated kinase Ste20 to its downstream substrate Ste11, which will then initiate a phosphorylation cascade that leads to changes in gene expression and cell morphology required for mating. (D) Schematic representation of the domain rearrangement library. Each gene encoding more than one domain was split respecting domain boundaries and all possible recombinations were done as represented by blue lines (GenBank accession numbers for individual domains used to construct the library are listed in Data S3) [11]. (E) Subsets of the rearrangement library corresponding to all proteins containing at least one domain from a given gene (i.e., Ste11 in the example) were then transformed into a strain in which the corresponding gene (Ste11 in the example) had been deleted, thus replacing the WT gene with a library of domain rearrangement variants that include at least one domain from the deleted gene.

While in nature domain rearrangements can occur by a variety of mechanisms [4],[5],[7],[8],[12], in principle two major outcomes are possible: a protein with a new domain combination is created, without altering pre-existing genes (Figure 1A), or the creation of a protein with a new domain combination concomitantly replaces a protein with a pre-existing domain combination (Figure 1B). Recently, it has been shown that signaling network function can be altered, when domain-rearrangement events create proteins with new domain combinations without replacing pre-existing genes [11]. While this work demonstrated that domain rearrangements could be a major force in the evolutionary diversification of signaling pathways, a far more challenging question still needs to be addressed: How are domain rearrangements tolerated when the genetic mechanisms involved result in the simultaneous replacement of a pre-existing gene? Understanding how these replacements are tolerated is difficult if one considers that not all of the pre-existing functions are preserved, and that the proteins involved are often part of large multi-protein complexes believed to have defined 3D structures, and thus likely to impose spatial constraints. Furthermore, it has been postulated recently that, because of the possible combinatorial complexity involved in the assembly of multi-protein complexes, signaling complexes within a cell might have compositional heterogeneity [21]–[23]. Thus, to fully comprehend how domain-rearrangements may affect signaling networks function, it is also necessary to understand the mechanisms by which the concomitant replacements are tolerated. This knowledge would advance our understanding of fundamental aspects of network evolution and, as importantly, could enable the development of efficient tools for signaling engineering.

To answer this question, we used a synthetic biology approach to systematically determine the robustness of the mitogen activated protein kinase (MAPK)-mediated yeast mating pathway (described in Figures 1B and S1) [24] to 92 domain-rearrangement events that replace 11 pre-existing genes. Specifically, we created a library in which 22 domains from 11 mating pathway proteins were shuffled (as described in [11] and shown in Figure 1C). Library variants in which domains from a given protein (e.g., the MAP3K Ste11 in the example in Figure 1D) were shuffled with domains from all other proteins, were transformed into a yeast strain in which the corresponding gene (Ste11 in the example) had been previously deleted. In this manner, we effectively replaced a wild type (WT) gene with all library constructs that include at least one domain from the replaced gene. By repeating this procedure for individual deletion strains in which either the G protein β subunit Ste4, the G protein γ subunit Ste18, the scaffold Ste5, the adaptor Ste50, the PAK kinase Ste20, the MAP3K Ste11, or the MAP2K Ste7 had been deleted, we generated a library of strains in which a WT gene has been replaced by all domain-rearrangement mutants involving domains from that gene (Figure S2). The mutational mechanisms that rearrange domains in natural proteins are obviously different from the two-part shuffling method used to construct our library. However, we are not interested here in investigating specific mechanisms leading to domain rearrangement, but rather the functional consequences that these rearrangements have at the protein and network level. Furthermore, while evolution could rearrange domains from any pair of proteins in the genome (though recent evidence suggests that rearrangements can preferentially occur among functionally related genes [25]), for simplicity we limited our analysis to rearrangement events between proteins belonging to the mating pathway. Assessing how general the results presented here are would require a genome-wide analysis that is beyond the scope of this work.

## Results

### The Mating Signaling Pathway Is Robust to Domain Rearrangement-Mediated Replacements

**Figure 2:**
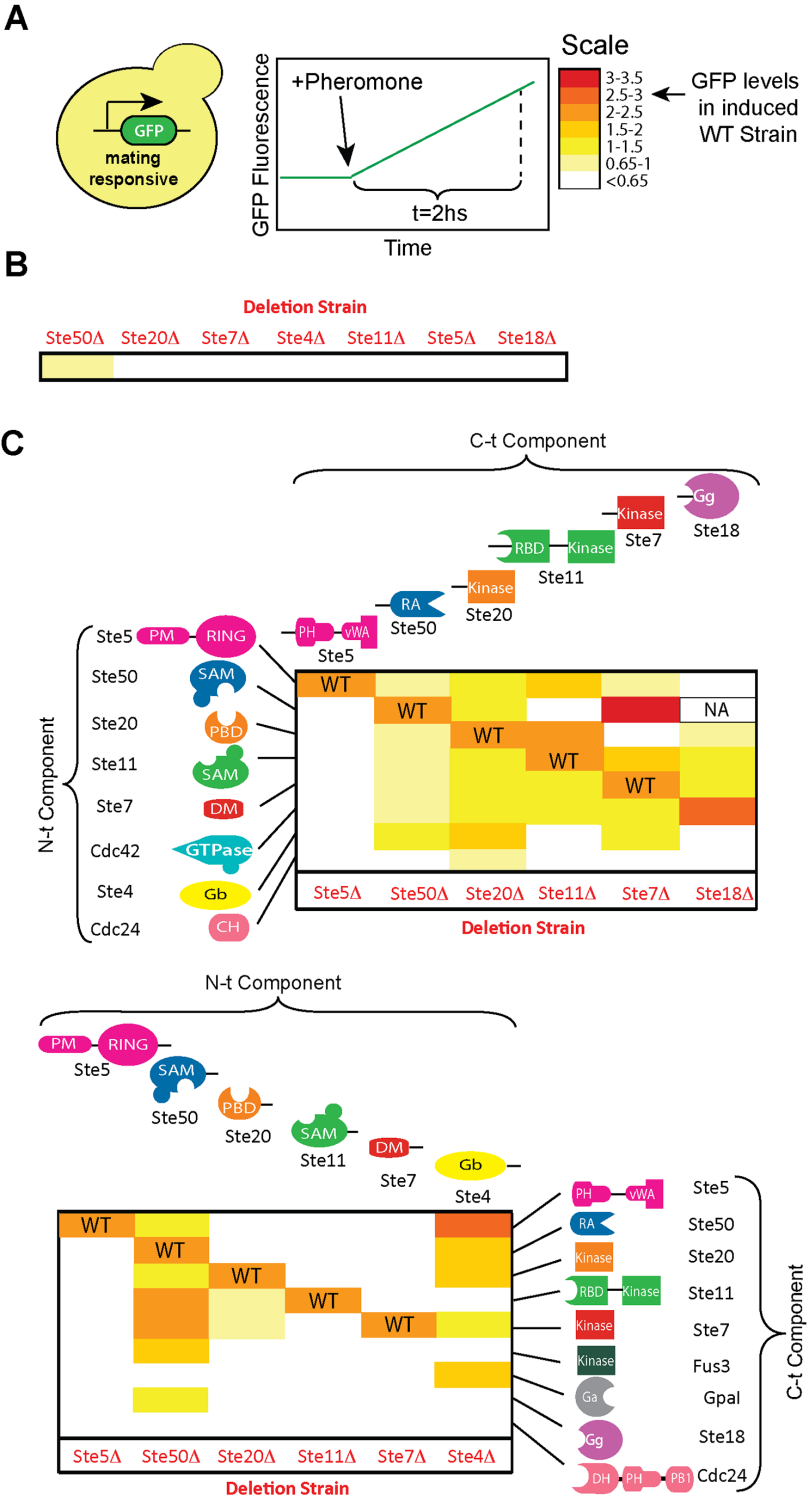
The yeast mating pathway is robust to domain rearrangement-mediated gene replacements. (A) Mating pathway activation was determined by flow cytometry, measuring the fluorescence intensity of a GFP reporter controlled by a mating-responsive *FUS1* promoter, 2 hours after addition of 1 µM α-factor. (B) As expected, individual deletions of the pathway components Ste50, Ste20, Ste7, Ste4, Ste11, Ste5, or Ste18 eliminate pathway activation [51],[52]. (C) Mating pathway activation for the library of domain rearrangement variants expressed in individual deletion strains. Rearrangement events that recreate WT genes are marked as “WT.” Repeated attempts to transform variant Ste50[N]-Ste18[C] failed, suggesting that it may result in cell toxicity. For a statistical analysis of the results see Figure S4. Data shown in Data S1.

To determine how domain-rearrangement mutations that replace pre-existing genes affect network function, we measured by flow cytometry the fluorescence levels of a green fluorescent protein (GFP) reporter controlled by a mating-responsive *pFUS1* promoter, before and 2 hours after stimulation with 1 µM mating pheromone (Figure 2A). As a control, we first confirmed that deletion of each individual gene abolishes pathway activation (with the exception of Ste50Δ that can still mediate very low, though statistically significant pathway activation, *t*-test, *p*≤0.013) (Figure 2B). Remarkably, we observed that 34 out of the 92 tested domain-rearrangement variant strains rescued pathway activation in a pheromone-dependent manner, above the levels observed in the corresponding deletion strains (Figures 2C and S3, with statistical analyses shown in Figure S4). In addition to changes in gene expression, mating pathway activation induces polarized growth that results in cell-cell fusion. To determine whether the tolerated rearrangement-derived replacements could also mediate polarized growth and cell-cell fusion, we determined the presence of pheromone-induced shmoos by microscopy and performed also quantitative mating assays. As shown in Figure S5, strains expressing active variants are capable of polarized growth. Furthermore, as shown in Figure S6, about 75% of the tested variants mate with at least 10% of the WT efficiency, while among those, ∼30% mate as efficiently as WT (for most variants, GFP-expression levels and mating efficiency correlate) (Figure S7). Taken together, our results demonstrate that domain rearrangement-mediated replacements can be tolerated, in some instances with pathway activation levels indistinguishable from WT.

While the mating pathway seems capable of tolerating domain rearrangement-mediated replacements, it is possible that at least some of these replacements could have detrimental effects on other cellular processes, and thus their evolutionary relevance would be questionable. To investigate this possibility, we measured the growth rate (as a proxy for fitness) of the most active domain rearrangement variants (nine variants in four different deletion strains). As shown in Figure S8A, the growth rate of the domain rearrangement variants is equal to, or even higher than, the growth rate of the WT strain, indicating that, at least under the laboratory conditions tested, domain rearrangement does not affect fitness negatively. Furthermore, for the domain rearrangement variants that functionally replace the MAP3K Ste11 in the Ste11Δ strain, we also measured growth rate under high osmolarity-induced stress, as in addition to mediating the mating response, Ste11 is also a MAP3K in the high osmolarity pathway [26]. As shown in Figure S8B, growth rates are not negatively affected by domain rearrangements involving Ste11, again suggesting that they do not impair fitness under the tested laboratory conditions.

While it is likely that some domain combinations would be unable to fold and/or function properly, there is no simple correlation between a domain rearrangement variant expression level and its ability to mediate mating pathway response (Figure S13). More likely, analysis of the data in Figure 2C reveals that while some components of the signaling network are essential, other are interchangeable. The integrity of the Ste5 scaffold seems critical for pathway function, as domain-rearrangement events involving Ste5 domains are never tolerated. Similarly, kinase domains cannot be replaced even by other kinase domains (e.g., replacement of the MAP2K Ste7 kinase domain by those of the PAK Ste20, MAP3K Ste11, or MAPK Fus3, failed to rescue pathway activity in the Ste7Δ strain). These results suggest that kinase-substrate specificities are firmly defined. In contrast, pathway function can be preserved when the N-terminal (N-t) interaction domains of the kinases Ste20, Ste11, or Ste7, responsible for localizing the kinase domains to the signaling complex, are replaced with alternative interaction domains. Thus, we hypothesized that the ability of the network to utilize alternative mechanisms of kinase recruitment to the signaling complex may contribute to network robustness against domain rearrangement-mediated replacements.

### Kinases Can Be Recruited to the Signaling Complex by Alternative Interaction Domains

**Figure 3:**
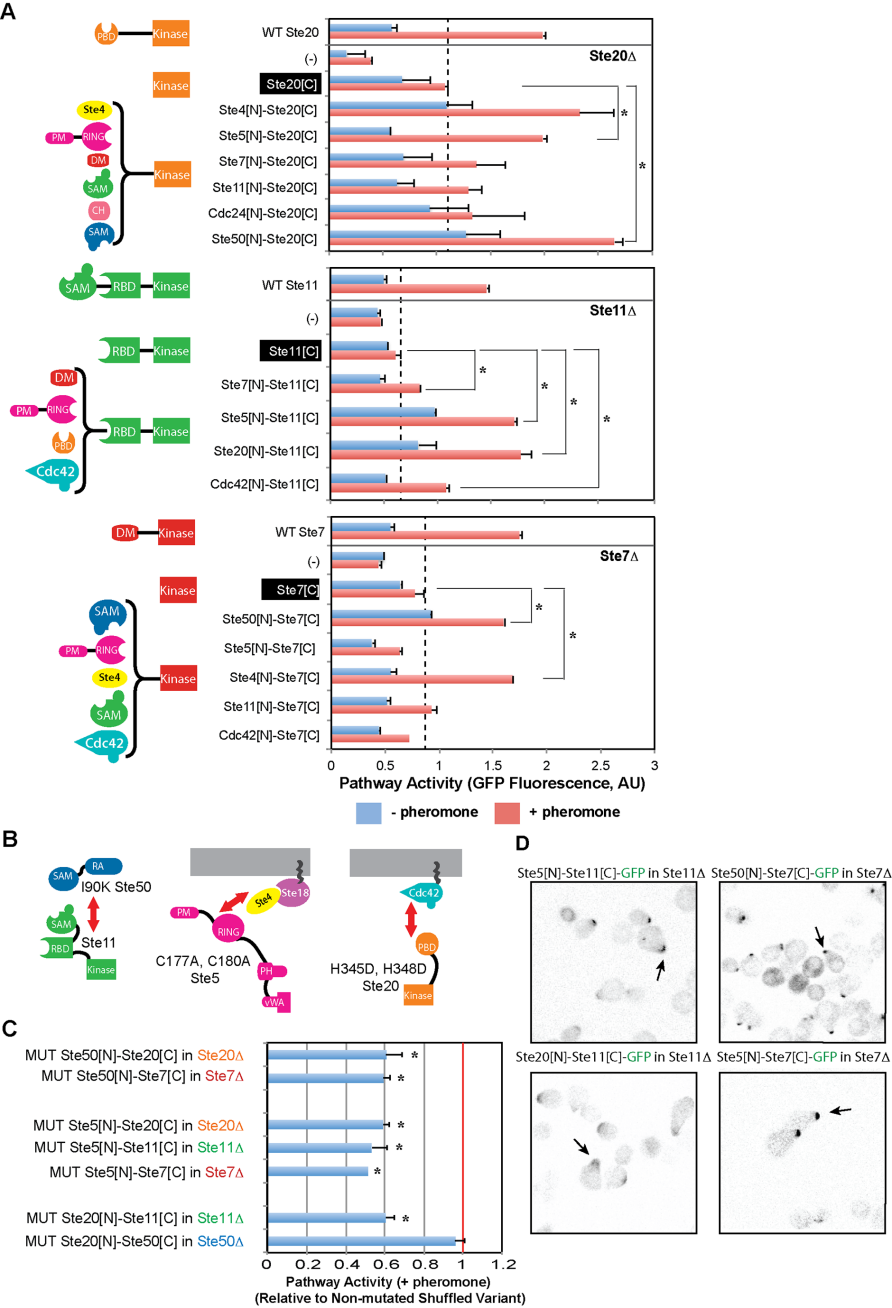
Kinases are recruited to the signaling complex by alternative N-t localization domains. (A) Comparison of mating pathway activation by kinase variants with or without N-t localization domains. In all cases, deletion of the full length kinase gene results in pathway inactivation. Expression of kinase variants lacking N-t localization domains only recovers partial pathway activation. In contrast, rearrangement events that fuse N-t domains known to interact with diverse partners in the mating signaling complex to the C-t kinase domains restore pathway activation to higher levels. (B) Schematic representation of the mutations introduced in Ste50 SAM domain, Ste5 RING domain, and Ste20 PBD domain. (C) Pathway activity for domain rearrangement variants carrying the mutations shown in (B) relative to the activity of the corresponding non-mutated variants. In most cases, mutations that disrupt specific recruitment interactions decrease pathway activation between 40%–50%. MUT Ste20[N]-Ste50[C] might still localize to the signaling complex, as Ste50's RA domain binds Cdc42 independently of Ste20's PBD domain [53]. (D) Fluorescence microscopy of GFP-tagged domain rearrangement variants shows that kinases can be recruited to the mating shmoo using alternative interaction domains. Statistically significant differences are marked with asterisks. Data shown in Data S1.

To explore this hypothesis, we first compared mating pathway activation mediated by the domain-rearranged kinase variants with those of kinase variants lacking N-t localization domains. As shown in Figure 3A (and further analyzed in Figures S9 and S10), variants lacking N-t localization domains activate pathway response very poorly, as compared to kinase variants with rearranged N-t domains. Second, we introduced in the N-t localization domains mutations that had been shown to reduce binding affinity with their respective interaction partners. Specifically, we mutated the following residues (Figure 3B): I90K in Ste50's N-t SAM domain, known to reduce binding to Ste11's N-t SAM domain [27]; C177A and C180A in Ste5's RING domain, known to reduce binding to the Gβ Ste4 [28]; and H345D H348D in Ste20's PBD domain, known to reduce binding to the small GTPase Cdc42 [29]. As shown in Figure 3C, in seven out of eight cases, point mutations reduced pathway activation between 40%–60%, suggesting that the alternative N-t localization domains are effectively recruiting the kinases to the signaling complex. Finally, we further confirmed this hypothesis by fluorescence microscopy, using GFP-tagged domain rearranged variants. As depicted in Figure 3D, kinases with rearranged N-t interaction domains are still recruited to the mating shmoo, suggesting that they localize to the signaling complex (note that when GFP is expressed alone, it is uniformly distributed in the cytoplasm) (Figure S11). Thus, we conclude that the ability of the signaling complex to accommodate alternative mechanisms of kinase recruitment (Figure S12) contributes to the robustness of the network to domain rearrangement-mediated replacements.

### Disordered Inter-domain Linkers Are Not Required to Tolerate Domain Rearrangement-Mediated Replacements

**Figure 4:**
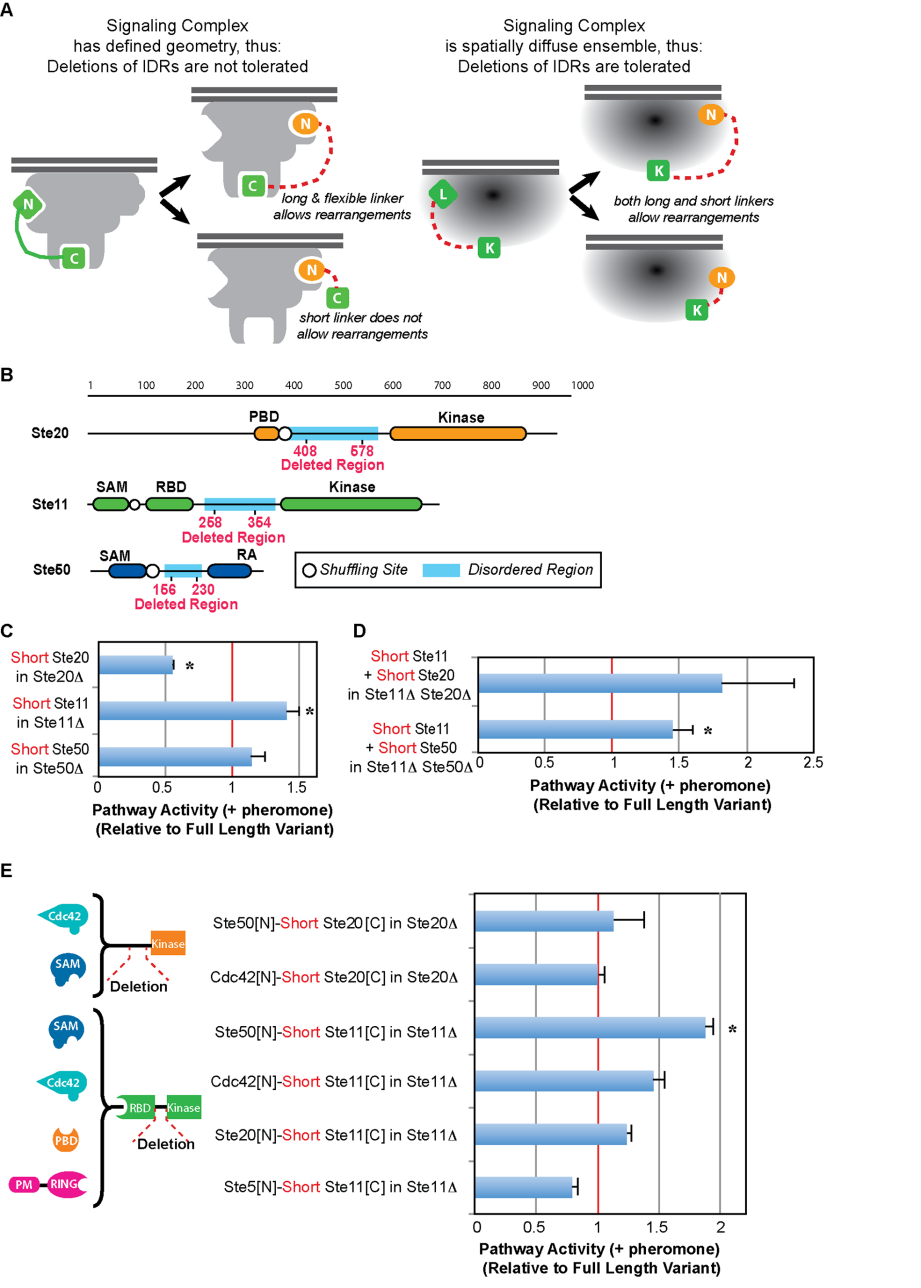
Exploring the mechanisms that enable signaling complexes to tolerate rearrangement-mediated gene replacements. (A) Differentiating between two alternative hypotheses: removal of IDRs should negatively impact signaling function if the signaling complex possesses well-defined spatial constraints and therefore a rather rigid structure (left). In contrast, removal of IDRs could be tolerated if the complex is flexible and can adopt a wide ensemble of conformations (right). (B) Schematic representation of the IDR deletion variants. (C) Mating pathway function in yeast strains with IDR-deleted Ste11, Ste20, or Ste50 variants (“Short”), relative to their corresponding full-length variants. (D) Mating pathway function in yeast strains with pairs of simultaneously IDR-deleted variants (either Ste11 and Ste20, or Ste11 and Ste50) in the respective double KO, relative to their corresponding full-length variants. (E) Mating pathway function in yeast strains with IDR-deleted domain rearrangement variants. Statistically significant differences are marked with asterisks. Data shown in Data S1.

We then investigated the mechanisms that enable the signaling complex to tolerate changes in recruitment interactions. Domain-domain interactions depend on specific binding interfaces, thus they are unlikely to tolerate drastic changes in interaction partners. Thus, if alternative recruitment has to maintain specific domain-domain interactions, two hypotheses are possible (Figure 4A): (i) rearrangements are tolerated because, even though signaling complexes possess precisely defined spatial constraints that result in fairly rigid 3D structures, domains are connected by long and flexible linkers (e.g., Ste5, Ste7, Ste11, Ste20, and Ste50 are predicted to contain intrinsically disordered regions [IDRs] [30] ranging from ∼74 to ∼207 amino acids long, within their inter-domain linkers, see Figure S14); or (ii) rearrangements are tolerated because signaling complexes do not possess rigid spatial constraints, but are rather diffuse ensembles of dynamically interacting proteins [31]–[33]. We reasoned that, if signaling complexes had defined spatial constraints and thus IDRs were required for networks to tolerate domain-rearrangement-mediated replacements, deletion of IDRs located within inter-domain linkers should substantially reduce pathway function. In contrast, if signaling complexes do not possess tight spatial constraints, but are rather loosely defined regions in which multiple weak interactions create higher local concentrations of signaling proteins, then deletion of inter-domain IDRs should not be detrimental to pathway function. To differentiate between these two hypotheses, we deleted segments of 171 amino acids from Ste20's IDR, 97 amino acids from Ste11's IDR, and 74 amino acids from Ste50's IDR and determined the ability of the shortened proteins to mediate pathway activation, as compared to their respective full-length variants. Note that we did not analyze IDRs present in Ste7's N-t and Ste5's N-t or C-terminus (C-t) because they do not connect pairs of domains, and thus are not likely to facilitate inter-domain flexibility. As shown in Figure 4C, all three shortened variants are still capable of mediating pathway activation (the decrease observed with Ste20's short variant is expected, as Ste20's IDR contains a proline-rich motif needed for proper binding of Bem1, a Cdc42 interaction partner, that when mutated has been shown to reduce pathway activation by ∼50% [34]). To further explore the role of IDRs in pathway function, we simultaneously replaced two WT proteins for their corresponding shortened variants. As shown in Figure 4D, co-expression of IDR-deleted Ste11 and IDR-deleted Ste20 variants effectively mediates pathway activation in the double deletion strain Ste20Δ Ste11Δ; similarly, co-expression of IDR-deleted Ste11 and IDR-deleted Ste50 variants effectively mediates pathway activation in the double deletion strain Ste50Δ Ste11Δ. These results indicate that the mating signaling complex can tolerate simultaneous deletions of IDRs in at least two proteins. Finally, we asked whether IDRs were necessary to tolerate domain rearrangement-mediated replacements, by measuring pathway activation for IDR-deleted domain-rearranged variants, as compared to their respective full-length variants. As shown in Figure 4E, deletion of the IDRs does not reduce pathway activity for most of the domain rearrangement variants tested, suggesting that IDRs are not needed to tolerate domain rearrangement-mediated replacements. Taken together, our results suggest that the yeast mating signaling complex does not possess a rigid, precisely defined spatial geometry, or that at least multiple alternative conformations are functional. Though not the focus of this study, we also noticed that, in some instances, deletion of the IDRs increased basal levels of pathway activation (Figure S15). This observation suggests that, while IDRs are not required for pathway function, they might have regulatory roles.

### The Mating Signaling Pathway Tolerates Simultaneous Changes in Multiple Domain-Mediated Interactions

**Figure 5:**
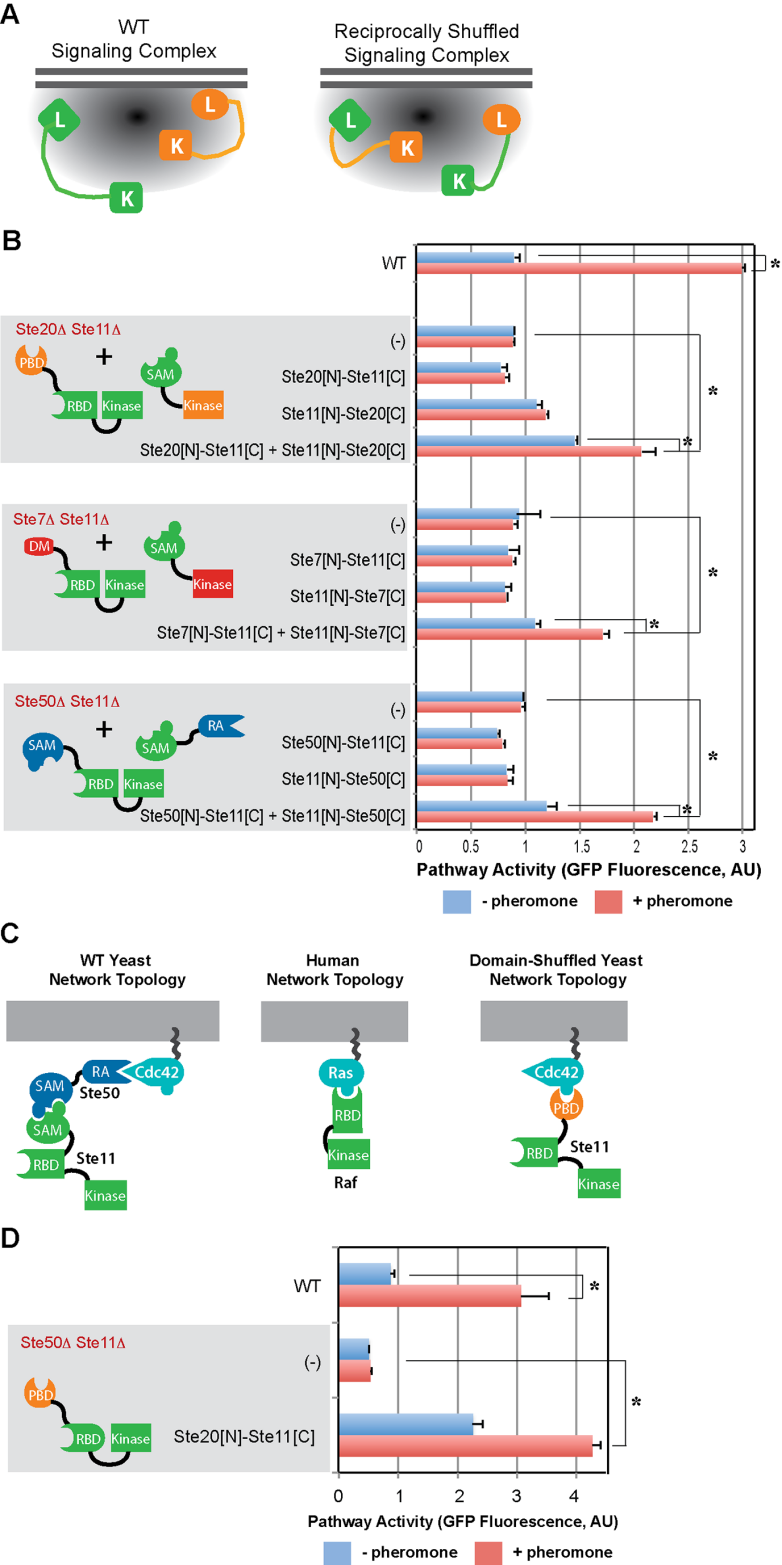
Signaling complexes can tolerate multiple rearrangement-mediated gene replacements. (A) Schematic representation of the reciprocally rearranged variants. (B) Co-expression of Ste20[N]-Ste11[C]+Ste11[N]-Ste20[C] restores pathway activation in the Ste20Δ Ste11Δ strain, co-expression of Ste7[N]-Ste11[C]+Ste11[N]-Ste7[C] restores pathway activation in the Ste7Δ Ste11Δ strain, and co-expression of Ste50[N]-Ste11[C]+Ste11[N]-Ste50[C] restores pathway activation in the Ste50Δ Ste11Δ strain. (C) Changes in network topology resulting from domain rearrangement events in our experiments, mimic changes in network topology that have occurred during evolution. (D) Expression of the domain rearranged variant Ste20[N]-Ste11[C] in the double deletion strain Ste50Δ Ste11Δ rescues pathway activation. Statistically significant differences are marked with asterisks. Data shown in Data S1.

We hypothesized that if signaling complexes possess loosely defined spatial constraints, the network should tolerate more complex domain rearrangement events, such as those in which domains from pairs of proteins are reciprocally rearranged (Figure 5A). To test this hypothesis, we introduced the pairs of reciprocally rearranged variants Ste20[N]-Ste11[C]+Ste11[N]-Ste20[C], Ste7[N]-Ste11[C]+Ste11[N]-Ste7[C], and Ste50[N]-Ste11[C]+Ste11[N]-Ste50[C], in the double deletion strains Ste20Δ Ste11Δ, Ste7Δ Ste11Δ, and Ste50Δ Ste11Δ, respectively. As shown in Figure 5B, while transformation with any of the single domain rearrangement variants did not rescue the double deletions, transformation with each pair of reciprocally rearranged variants rescued pathway activation, demonstrating that the mating signaling complex can accommodate changes in domain connectivity in two components simultaneously, supporting the hypothesis that the signaling complex does not possess a rigid, precisely defined geometry.

### The Synthetic Domain Rearrangement Variants Analyzed in Our Experiments Resemble Naturally Evolved Proteins

Some of the changes in network topology resulting from domain rearrangement events in our experiments mimic changes in network topology that have occurred during evolution. For instance, in yeast, the adaptor Ste50 mediates the interaction between the MAP3K (Ste11) and the small GTPase (Cdc42) (Figure 5C). In contrast, in humans the adaptor Ste50 has been lost and, instead, there is a direct interaction between the MAP3K Raf and the small GTPase Ras [35]–[38]. The domain rearrangement variant Ste20[N]-Ste11[C] topologically resembles human Raf, as the N-t Ste20 PBD domain interacts with Cdc42 directly (in Raf this interaction is mediated by the RBD domain, but Ste11's RBD binds Ste5). To test the hypothesis that Ste20[N]-Ste11[C] could functionally resemble Raf, we measured the ability of the domain rearrangement variant Ste20[N]-Ste11[C] to mediate pathway activation in a strain in which both Ste50 and Ste11 had been deleted. As predicted, expression of Ste20[N]-Ste11[C] complements the simultaneous loss of Ste50 and Ste11 (Figure 5D), confirming that the network topology evolved in our experiment functions similarly to the network evolved in metazoans.

Finally, among the seven kinase-containing domain combinations that in our experiments resulted in active pathways (Figure S16A), three have not been previously found in yeast mating pathway proteins (e.g., domain combinations in which Cdc42's small GTPase domain, Ste5's RING domain, or Ste4's β-propeller domain, are connected to kinase domains). However, as these domain combinations lead to functional proteins in our model system, we hypothesized that proteins with similar domain combinations are likely to be found in natural genomes. To explore this hypothesis, we searched the Domain Club Database [10] to identify natural proteins with domain combinations resembling those found in our experiments. As shown in Figure S16B, we identified the human proteins: (i) LRRK1/2, with a domain composition that includes both small GTPase and kinase domains; (ii) PIK3R4 (a regulatory subunit of the PI3K complex) with a domain composition that includes both β-propeller and kinase domains; and (iii) MAP3K1, with a domain composition that includes RING and kinase domains. Thus, while the functions of these proteins need not be related to those in our experiments, these results indicate that the novel domain combinations that lead to active proteins in our screening have also evolved naturally.

## Discussion

Our results indicate that the yeast mating pathway is remarkably robust to domain rearrangement-mediated replacements, tolerating multiple changes in recruitment interactions. In particular, we observed that the N-t domains or motifs of the three multi-domain kinases in the mating pathway (Ste20, Ste11, and Ste7), which normally localize the respective kinase domains to the mating signaling complex, can be replaced by alternative interaction domains (from other kinases, or from other pathway components). In contrast, kinase domains cannot be replaced, suggesting that the specificity of kinase-substrate interactions is key for proper signaling function. Thus, while the inter-molecular connectivity of the domains is important, the intra-molecular connectivity is not as important, suggesting that proper network function depends more on the formation of a signaling complex composed of key domains, rather than key proteins. Interestingly, even though intra-molecular interactions between different domains within a protein may play regulatory roles [17], we observed that for most domain rearrangement variants, the basal levels of pathway activation are similar to, or only slightly higher than those of the WT pathway (Figure S3). This may simply reflect the fact that activation of Fus3, the bottom kinase in the pathway, requires two concurrent stimuli: (i) phosphorylation-dependent activation of the MAP2K Ste7, and (ii) pheromone-dependent activation of the mating scaffold Ste5 [39]. Thus, even if domain rearrangement altered intra-molecular regulation and therefore increased the activity of upstream mating kinases, signal propagation would still depend on phosphorylation-independent activation of Ste5.

Because domain-domain interactions are highly specific, proteins with rearranged domain compositions may have altered localization within the signaling complex. How can then domain rearrangements be tolerated? Initially, we hypothesized that the presence of long, disordered inter-domain linkers may enable each domain within a rearranged protein to localize to the correct site within the complex. However, we found that the IDRs present within inter-domain linkers are dispensable for pathway function and, more importantly, for the robustness of the network to domain rearrangements. Taken together, these observations suggest that the function of the mating signaling complex is not constrained by a defined geometry. Thus, we propose that, rather than a precisely assembled multi-molecular machine, the yeast mating signaling complex is an ensemble of dynamically interacting molecules with loose spatial constraints [40],[41]. A “tridimensional meshwork,” in which individual components are only transiently bound by multiple, weak interactions, makes sense if one considers that mating signaling complexes should be able to rapidly re-orient to follow changes in the direction of the pheromone gradient, as well as to accompany the growth of mating projections. Similar matrix-like meshworks have been postulated to explain the dynamic nature of microtubule plus-end tracking proteins, which rapidly track microtubules movement [42]. Furthermore, recently Mayer, Deeds, and their co-workers have postulated that, because of the combinatorial complexity involved in the assembly of multi-protein complexes, rather than a single complex with a defined composition, it is more likely that multiple complexes with different compositions might exist simultaneously [21]–[23]. Remarkably, Suderman and coworkers [23] computationally modeled the yeast mating pathway and showed that the mating signal could still be propagated by compositionally heterogeneous populations of complexes. Our results suggest that signaling complexes are not only compositionally heterogeneous, but also structurally flexible.

While in the short term mutational robustness buffers the impact that genotypic changes could have on phenotype, in the long term, mutational robustness may facilitate evolution [43],[44]. In particular, and as best described by Gerhart and Kirschner in their theory of facilitated variation [45], mutational robustness may enable the network to explore regions of genotypic space that, though presently neutral, could lead to adaptation in the event of future environmental or genetic changes [46],[47]. The relaxed spatial constraints of the mating signaling complex may enable the network to tolerate changes in protein interactions that result from the mutational events that lead to domain rearrangements. While still hypothetical, one could imagine that proteins with altered domain compositions may eventually evolve novel functions [10].

As affordable genome and transcriptome sequencing are rapidly expanding the list of domain-rearrangement mutations involved in disease [48], our work may help understand how disease-causing mutations affect the function of signaling complexes with components homologous to those analyzed in this work. Finally, the fundamental principles revealed here suggest that flexible multi-protein complexes could be ideal targets for cellular engineering [20].

## Materials and Methods

### Yeast Strains

Deletion strains were derived from a W303 strain with the following genotype: *MATa, bar1::NatR, far1Δ, mfa2::pFUS1-GFP, his3, trp1, leu2, ura3*. Seven strains were created in which the following genes from the mating pathway were deleted individually: Ste4, Ste5, Ste7, Ste11, Ste18, Ste20, and Ste50, in all cases using Trp as a selectable marker. Deletion strains were validated by genomic PCR and flow cytometry (each individual deletion of a “Ste” gene impaired pathway-dependent GFP expression). Double deletion strains (Ste20Δ Ste11Δ, Ste7Δ Ste11Δ, and Ste50Δ Ste11Δ) were also made by homologous recombination, using Leu as the second selectable marker.

### Domain-Rearrangement Libraries

The domain-rearrangement libraries were designed and constructed using a previously described combinatorial cloning strategy [11]. All variants were expressed from centromeric plasmids with Leu selection, under control of a constitutive low expression promoter consisting of a 250-bp fragment of the *CycI* promoter, and an AdhI transcription terminator.

### Flow Cytometry

Each strain carrying an individual deletion (or a double deletion, as in Figure 5) was transformed with a domain-rearrangement variant (or a combination of two, as in Figure 5) that effectively replaced the deleted gene(s). Samples were induced with 1 µM α-factor (Zymo Research), while controls were left untreated. Cultures were grown for two more hours, upon which protein synthesis was stopped by addition of cyclohexamide. GFP fluorescence was measured by flow cytometry, using a Miltenyi MACSQuant VYB flow cytometer. The GFP signal was averaged for all duplicates and standard errors were calculated. All experiments were repeated at least twice (total number of colonies analyzed: *n*≥4) and found to be in good agreement.

### Statistical Analyses

Two tailed *t*-tests with unequal variances were performed to assess the statistical significance of the differences in GFP fluorescence values measured by flow cytometry for the different samples.

### Fluorescence Microscopy

All domain-rearrangement variants were tagged with GFP at their N-termini, as previously described [11]. Imaging was performed with an automated inverted Leica TCS SP8 confocal microscope.

### Quantitative Mating Assays

Mating assays were performed with minor modifications to a previously described method [11]. Specifically, each “a-type” individual deletion strain (SO992, W303-derived, *trp1, leu2, ura3, his3, ADE2 can1*) described above was transformed with appropriate plasmids encoding each domain-rearrangement variant to be tested. Equal amounts of “A-type” cells transformed with each variant (or controls) were mixed with WT “α-type” cells and deposited on the surface of a polycarbonate filter placed on a YPD plate and incubated for 3 hours at 30°C. Cells were then detached from the filters by vortexing and aliquots were plated on minimum synthetic media, or synthetic media lacking lysine. Mating efficiency was calculated as the number of colonies on minimum synthetic media divided by number of colonies on synthetic media lacking lysine [49]. Results were normalized according to the WT type strain. Averages from triplicates and standard errors were calculated. The experiments were repeated at least twice (total number of colonies analyzed: *n*≥6) and found to be in good agreement.

### Site-Directed Mutagenesis

Site-direct mutagenesis was done by Quick Change, following the manufacturer's protocol (Quick Change II Site-Directed Mutagenesis kit, Agilent). Mutations were verified by DNA sequencing.

### Identification of Proteins in Natural Genomes with Domain Compositions Similar to Those Found in our Library Screening

Proteins with domain compositions similar to those found in our experiments were identified in the Domain Club Database [10].

### Estimation of the Volume Occupied by Intrinsically Disordered Regions

Hydrodynamic Radii for IDRs was calculated using the power law relation Rh = F * ρ_0_ * N^ν^ [50], where ρ_0_ is a constant that depends on persistence length, N is the number of residues in the polymer, ν is a scaling factor, and F is a correction factor that accounts for the net charge and Pro content of the IDR.

### Deletion of Intrinsically Disordered Regions

The IDRs of Ste50, Ste20, and Ste11 were identified in the Pfam database. Specifically, we deleted the disordered regions between amino acids 156 and 230 in Ste50, 408 and 578 in Ste20, and between 258 and 354 in Ste11.

## Supporting Information

Figure S1 **Function of each individual domain in the analyzed proteins.**
(TIF)

Figure S2 **Schematic representation of each subset of library variants transformed into the corresponding deletion strains (GenBank accession numbers for the sequences of all individual domains used to construct the libraries are listed in Data S3).**
(TIF)

Figure S3 **Basal levels of mating pathway activity (as determined by GFP fluorescence measured by flow cytometry).** GFP expression levels determined before addition of pheromone indicate that, in almost all cases, domain rearrangement-mediated gene replacements do not result in substantial constitutive activation of the mating pathway. Furthermore, even in those cases where basal pathway activation is higher than WT, addition of pheromone further increases GFP expression (compared data here with data on Figure 2C), indicating that pathways with domain-rearrangement replacements can be induced by pheromone. Data shown in Data S1 and Data S2.
(TIF)

Figure S4 **Statistical analysis of the GFP reporter measurements of mating pathway activation upon pheromone induction.** In all cases, the GFP values for pairs of variants were compared and the significance of the observed differences were assessed by performing two tailed *t*-tests. In (C) comparisons were made between the WT strain and strains carrying each domain rearrangement variant. If *p*>0.05 then we concluded that the GFP values measured for the corresponding variant are not significantly different from that measured for the WT. In (D) comparisons were made between each Δ strain and the corresponding strain carrying each domain rearrangement variant. If *p*<0.05 then we concluded that the GFP values measured for the corresponding variant are significantly different from that measured for the Δ strain. Data shown in Data S1.
(TIF)

Figure S5 **Domain rearrangement variants can mediate pheromone-induced polarized growth (“shmooing”).** Deletion strains carrying individual domain rearrangement variants were incubated for 1 h in the presence of 1 µM alpha-factor and representative images were taken using an automated inverted Leica TCS SP8 confocal microscopy, using a 63× objective. As controls, we included WT cells (able to shmoo), as well as strains carrying inactive domain rearrangement variants that, as expected, fail to induce polarized growth (two images at the bottom).
(TIF)

Figure S6 **Quantitative mating assays.** Mating assays: each “a-type” individual deletion strain was transformed with appropriate plasmids encoding each domain-shuffling variant to be tested. Equal numbers of “A-type” cells transformed with each variant (or controls) were mixed with WT “α-type” cells and deposited on the surface of a polycarbonate filter placed on a YPD plate, and incubated for 3 hours at 30°C. Cells were then washed from the filters and plated on minimum synthetic media or on synthetic media lacking lysine. Plates were incubated at 30°C for 48 hours and colonies on each plate were counted. Mating efficiency was calculated as the number of colonies on minimum synthetic media/number of colonies on synthetic media lacking lysine. Results were normalized according the WT strain. Averages from duplicates and standard errors were calculated. The experiments were repeated at least twice. Data shown in Data S2.
(TIF)

Figure S7 **Pathway activation, as determined by GFP fluorescence measured by flow cytometry 2 h after addition of 1 µM pheromone correlates, in most cases, with mating efficiency as determined in quantitative mating assays.** Data shown in Data S1 and Data S2.
(TIF)

Figure S8 **Determination of the growth rates for active domain rearrangement variants, as compared to the growth rates of the WT and the corresponding deletion strains.** Each strain was grown in liquid culture (in triplicates) and ODs were measured at 600 nm every hour for 8 hours. Data was fitted using the exponential equation: OD = OD_o_ e^λt^, where OD_o_ is the initial OD value, λ is the growth rate, and t is time. (A) Cultures were grown in rich media under isosmotic conditions. (B) Cultures were grown in rich media under high osmolarity stress (0.4 M KCl). Data shown in Data S2.
(TIF)

Figure S9 **Statistical analysis comparing the GFP values for the variants analyzed in** **Figure 3A** **, before or after addition of pheromone.** In all cases, two tailed *t*-tests were performed. Significant differences (*p*<0.05) are marked with asterisks. Data shown in Data S1 and Data S2.
(TIF)

Figure S10 **Ability of isolated N-t domains to mediate mating pathway activation.** The corresponding deletion strains were transformed with N-t localization domains alone and the fluorescence of the mating pathway reporter pFus1-GFP was measure before or 2 h after addition of pheromone. As shown in the figure, none of the localization domains can restore pathway activity in the deletion strains. Data shown in Data S2.
(TIF)

Figure S11 **The recruitment of the GFP-tagged variants to the mating projections seen in** **Figure 3D** **, depend on the presence of localization domains in the domain rearrangement variants.** When GFP is not fused to localization domains, it remains uniformly distributed in the cytoplasm, failing to localize to the mating shmoos.
(TIF)

Figure S12 **Alternative mechanisms of kinase recruitment to the signaling complex, for the domain shuffling variants shown to localize to the mating projections (shmoos) by fluorescence microscopy.** Note that our results suggest that the complex stoichiometry is flexible and can accommodate a diverse number of components.
(TIF)

Figure S13 **There is no simple correlation between a variant's expression levels and its ability to mediate mating pathway response.** Domain rearrangement variants were tagged at their N-t with GFP and transformed in the corresponding deletion strain (note that these strains did not have a mating reporter pFus1-GFP and therefore the only GFP signal measured was derived from the tagged variants themselves). GFP fluorescence was measured by flow cytometry. Data shown in Data S2.
(TIF)

Figure S14 **IDRs are found in Ste5, Ste7, Ste11, Ste20, and Ste50.** While in Ste7 and Ste5 IDRs are located at the protein termini, in Ste11, Ste20, and Ste50, IDRs are found within inter-domain linkers and, thus, they can separate folded domains by long and flexible distances. (A) Schematic representation of the IDR-containing mating pathway proteins Ste5, Ste7, Ste11, Ste20, and Ste50. IDRs are represented as light blue segments. (B) Estimation of the volume and maximum extended length of Ste11, Ste20, and Ste50 IDRs. (C) Schematic representation of the relative volume occupied by either IDRs or folded domains in Ste11, Ste20, and Ste50.
(TIF)

Figure S15 **Pathway activation (as determined by GFP fluorescence) for IDR-deleted variants, before the addition of pheromone, as compared with the corresponding full length variants.** In the single Δ strains, deletion of Ste20's or Ste50's IDRs does not affect basal levels of pathway activation. In contrast, deletion of Ste11's IDR causes small increases in basal pathway activation, suggesting it may alter the regulation of Ste11's kinase activity. Furthermore, in the double Δ strains, simultaneous deletion of Ste11 and Ste20 IDRs or of Ste11 and Ste50 IDRs leads to large increases in basal levels of pathway activation, suggesting that the simultaneous deletions have a marked effect in the regulation of pathway function. Statistically significant differences are marked with asterisks. Data shown in Data S1.
(TIF)

Figure S16 **Schematic representation of proteins with alternative domain combinations that lead to functional pathways in our experiments, and are also found in natural proteins.**
(TIF)

Data S1 **Data shown in** **Figures 2** **–** **5** **, and Figures S3, S4, S9, and S15.**
(XLSX)

Data S2 **Data shown in Figures S6–S8, S10, and S13.**
(XLSX)

Data S3 **GenBank accession numbers for sequences used to build the domain rearrangement library.**
(XLSX)

Text S1 **Supplemental material and methods.**
(DOCX)

## Abbreviations

GFP: green fluorescent protein
IDR: intrinsically disordered region
N-t: N-terminal
WT: wild type
