# Supplementary figures and images for: The Robustness of a Signaling Complex to Domain Rearrangements Facilitates Network Evolution
Source: PLoS Biol. 2014 Dec 9;12(12):e1002012. doi: 10.1371/journal.pbio.1002012 (PMC4260825; doi:10.1371/journal.pbio.1002012)

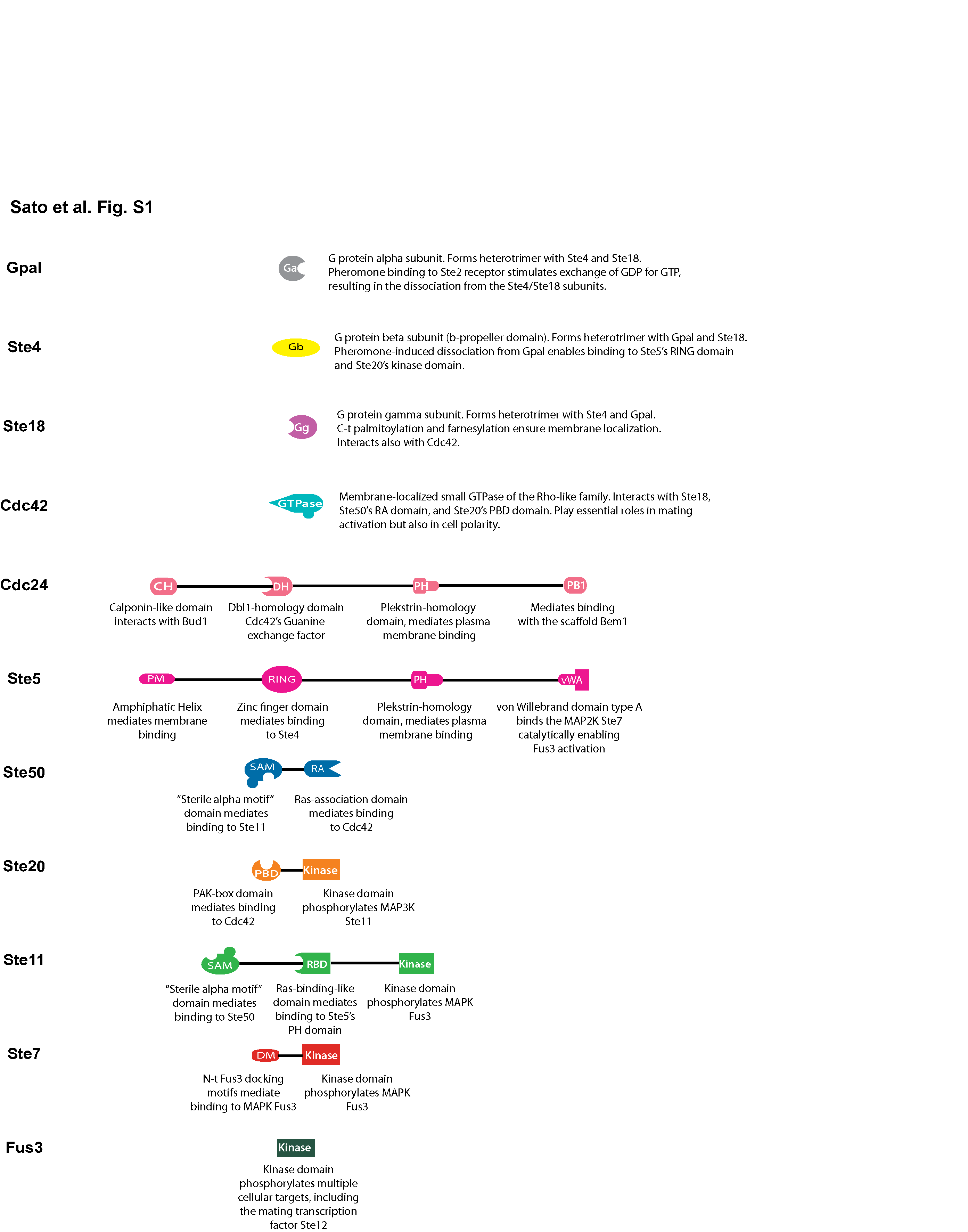

Supplement: Figure S1 — Function of each individual domain in the analyzed proteins. (TIF) [file pbio.1002012.s001.tif]

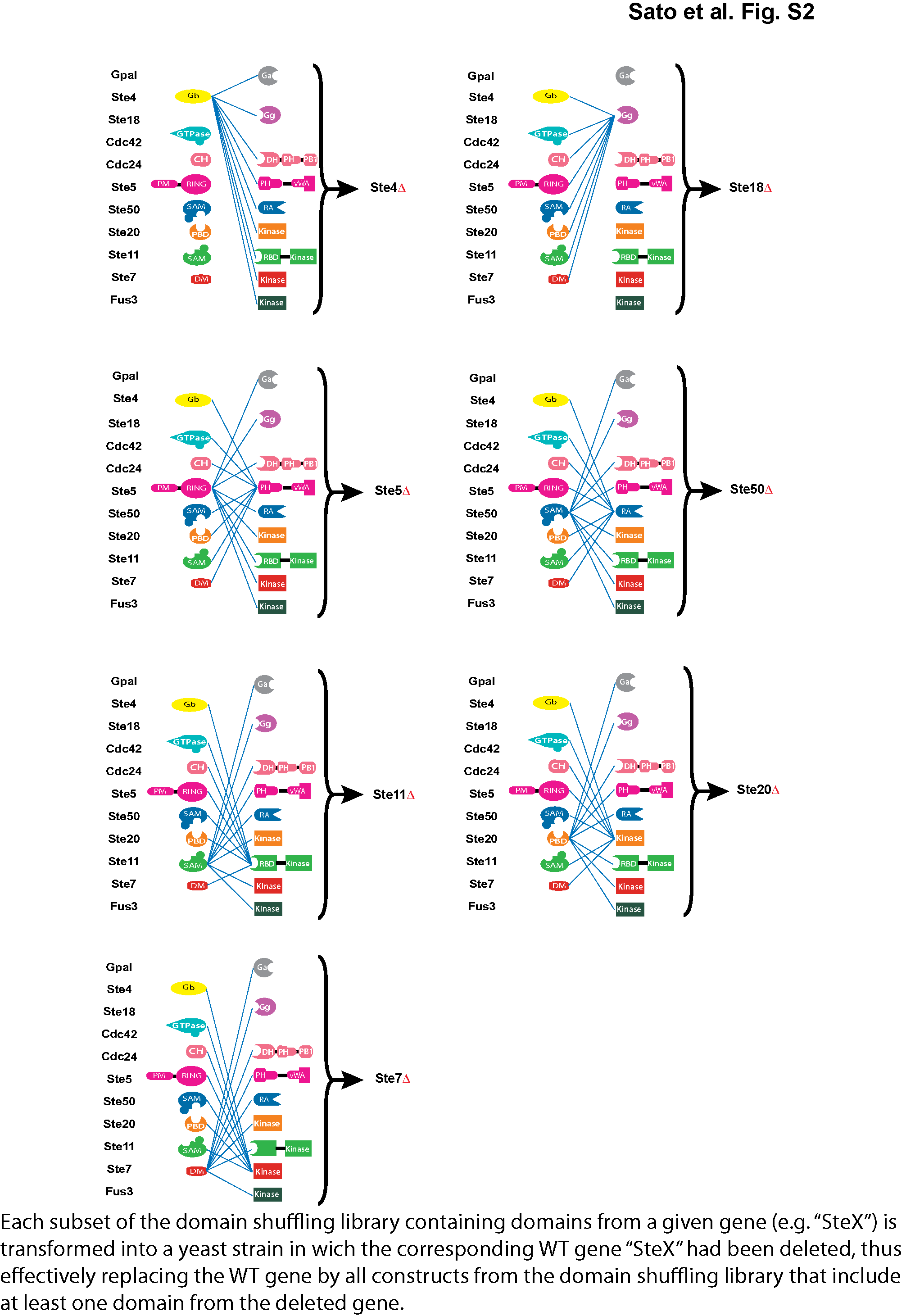

Supplement: Figure S2 — Schematic representation of each subset of library variants transformed into the corresponding deletion strains (GenBank accession numbers for the sequences of all individual domains used to construct the libraries are listed in Data S3). (TIF) [file pbio.1002012.s002.tif]

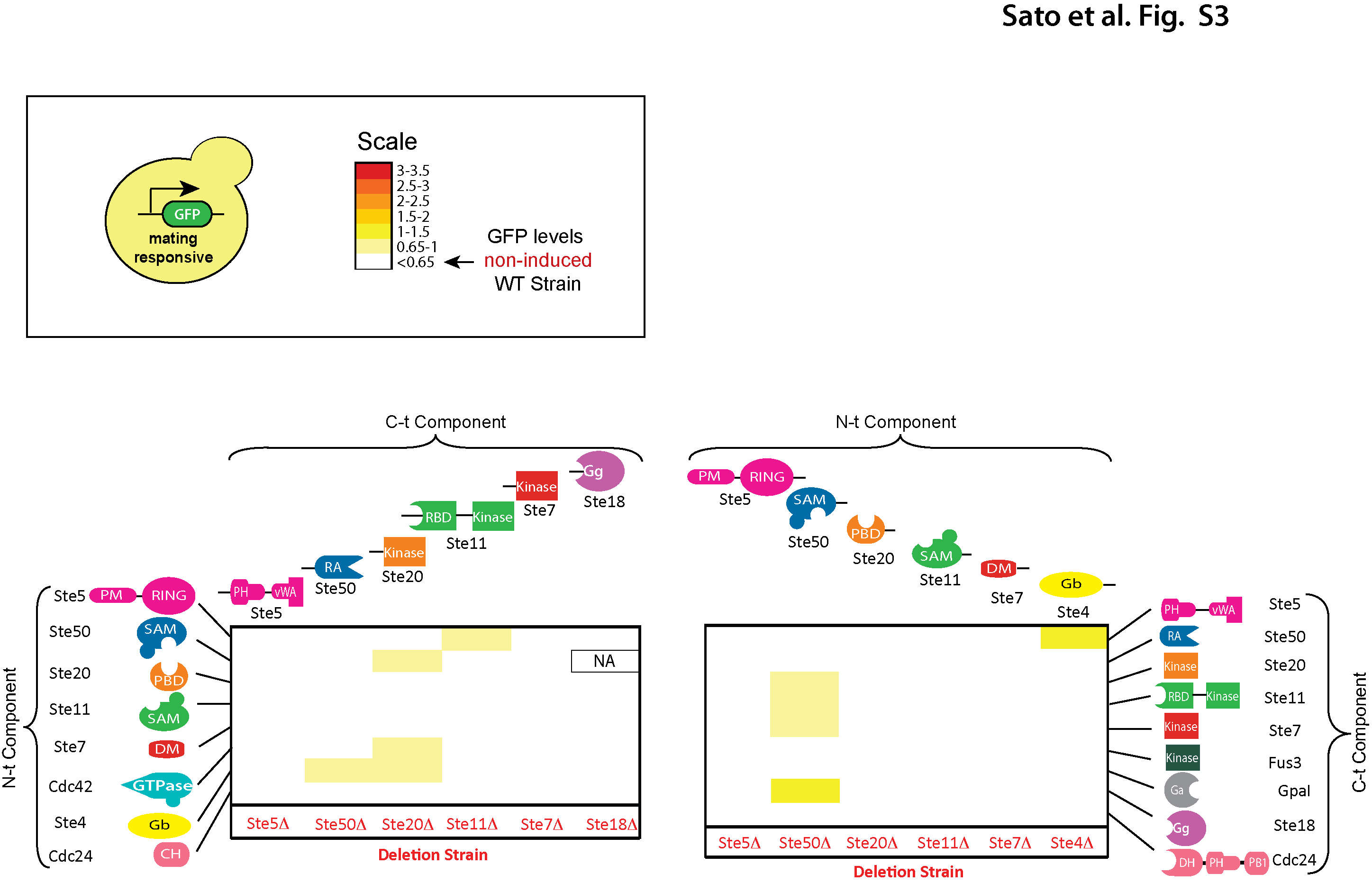

Supplement: Figure S3 — Basal levels of mating pathway activity (as determined by GFP fluorescence measured by flow cytometry). GFP expression levels determined before addition of pheromone indicate that, in almost all cases, domain rearrangement-mediated gene replacements do not result in substantial constitutive activation of the mating pathway. Furthermore, even in those cases where basal pathway activation is higher than WT, addition of pheromone further increases GFP expression (compared data here with data on Figure 2C), indicating that pathways with domain-rearrangement replacements can be induced by pheromone. Data shown in Data S1 and Data S2. (TIF) [file pbio.1002012.s003.tif]

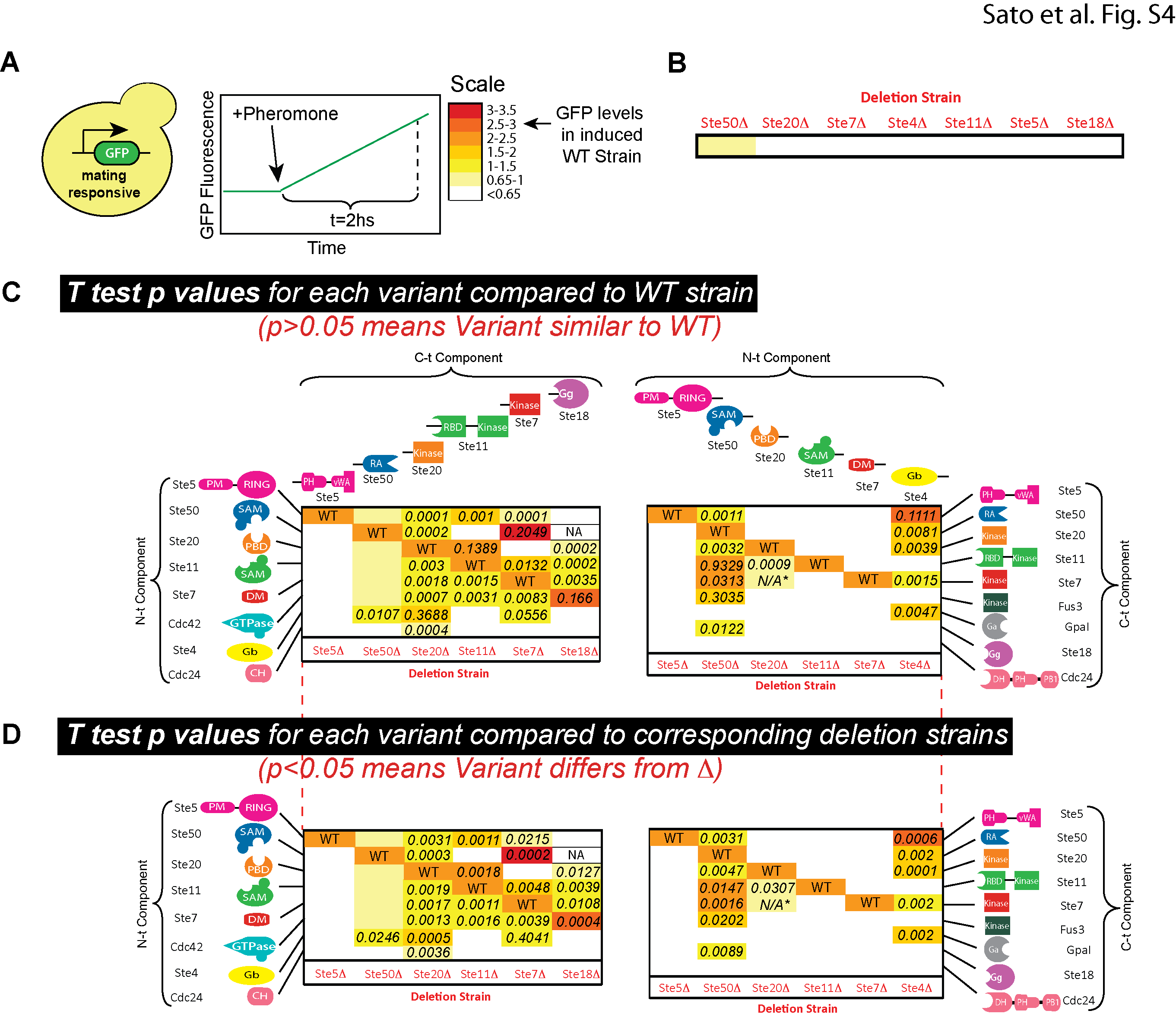

Supplement: Figure S4 — Statistical analysis of the GFP reporter measurements of mating pathway activation upon pheromone induction. In all cases, the GFP values for pairs of variants were compared and the significance of the observed differences were assessed by performing two tailed t-tests. In (C) comparisons were made between the WT strain and strains carrying each domain rearrangement variant. If p>0.05 then we concluded that the GFP values measured for the corresponding variant are not significantly different from that measured for the WT. In (D) comparisons were made between each Δ strain and the corresponding strain carrying each domain rearrangement variant. If p<0.05 then we concluded that the GFP values measured for the corresponding variant are significantly different from that measured for the Δ strain. Data shown in Data S1. (TIF) [file pbio.1002012.s004.tif]

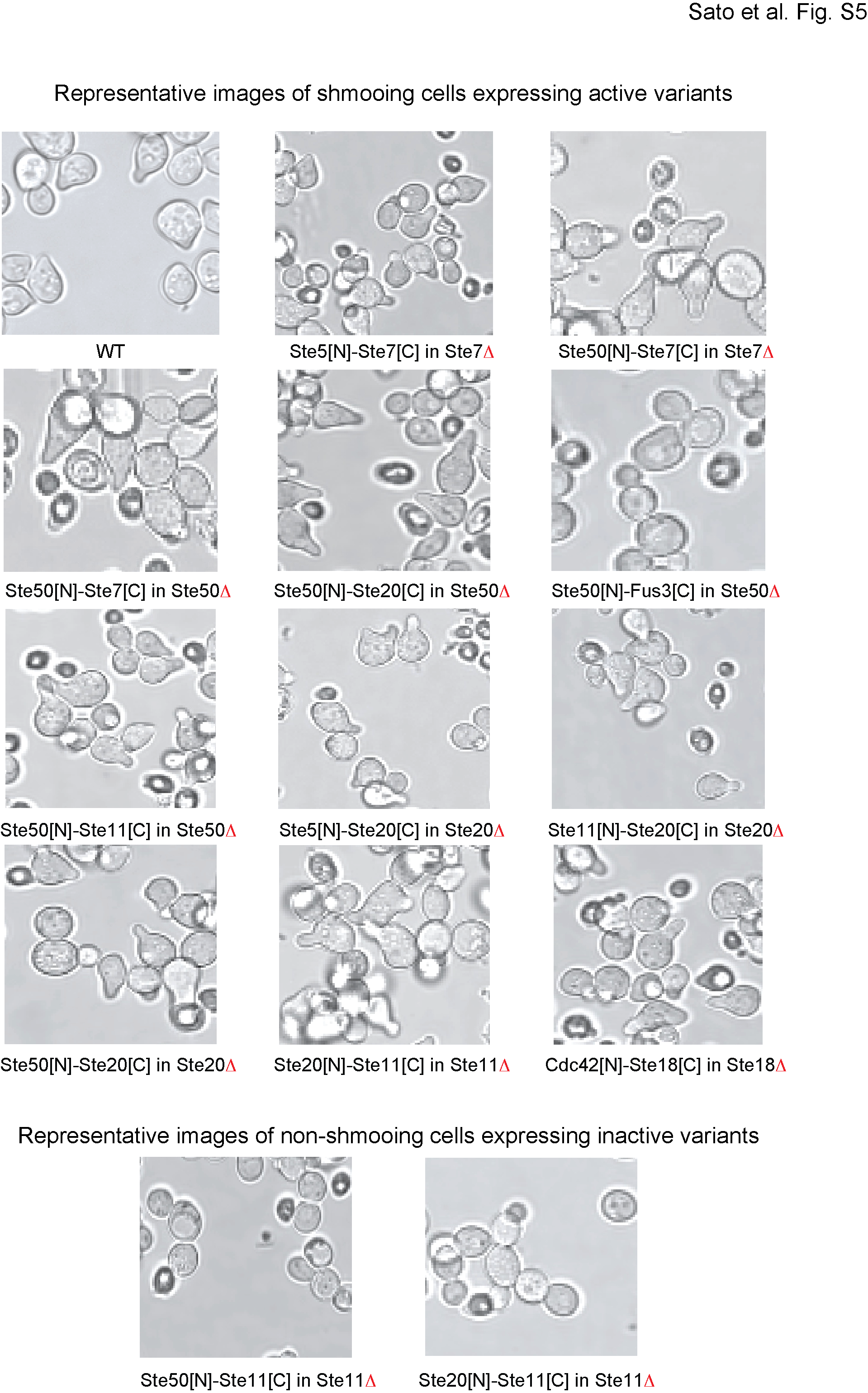

Supplement: Figure S5 — Domain rearrangement variants can mediate pheromone-induced polarized growth (“shmooing”). Deletion strains carrying individual domain rearrangement variants were incubated for 1 h in the presence of 1 µM alpha-factor and representative images were taken using an automated inverted Leica TCS SP8 confocal microscopy, using a 63× objective. As controls, we included WT cells (able to shmoo), as well as strains carrying inactive domain rearrangement variants that, as expected, fail to induce polarized growth (two images at the bottom). (TIF) [file pbio.1002012.s005.tif]

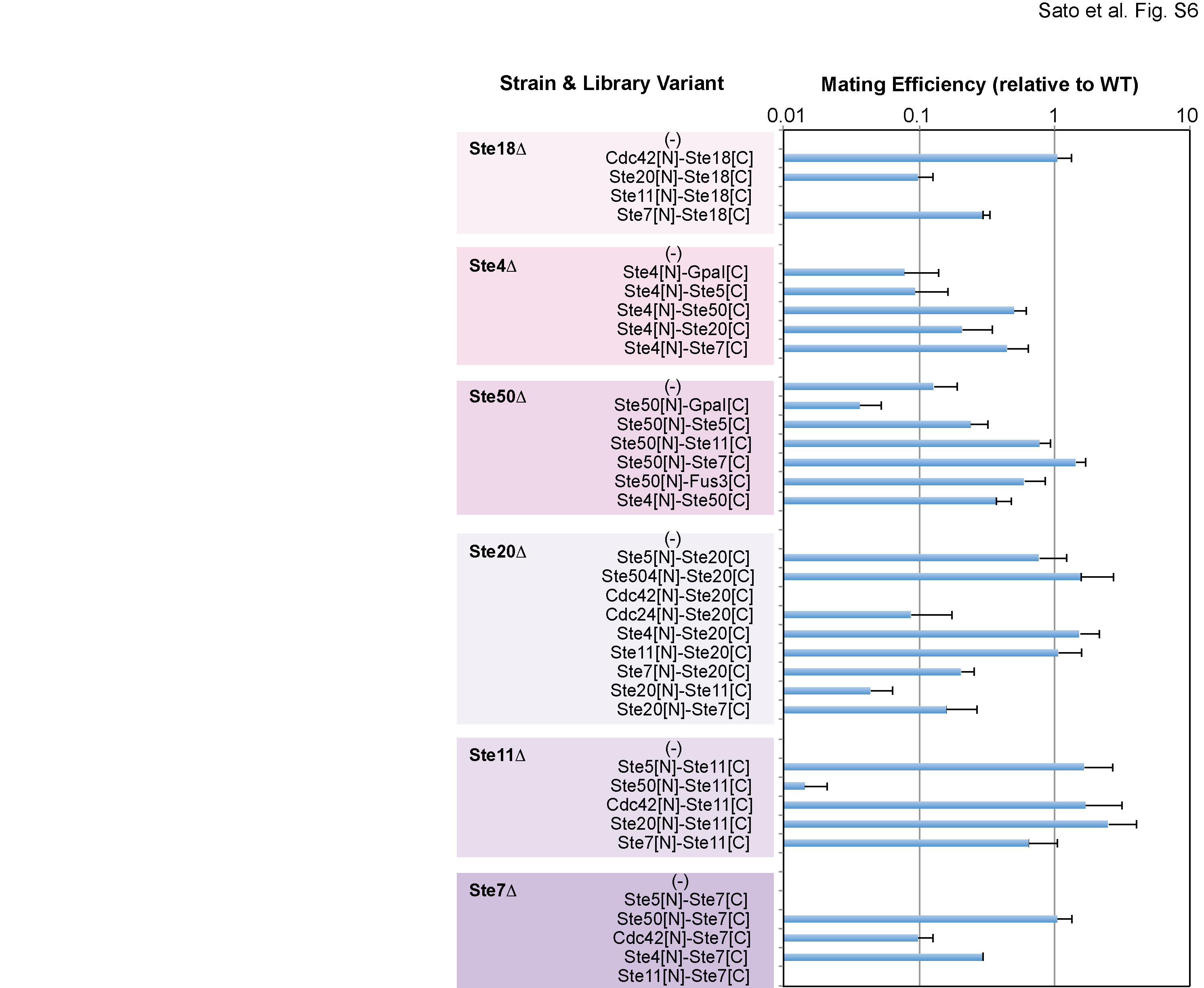

Supplement: Figure S6 — Quantitative mating assays. Mating assays: each “a-type” individual deletion strain was transformed with appropriate plasmids encoding each domain-shuffling variant to be tested. Equal numbers of “A-type” cells transformed with each variant (or controls) were mixed with WT “α-type” cells and deposited on the surface of a polycarbonate filter placed on a YPD plate, and incubated for 3 hours at 30°C. Cells were then washed from the filters and plated on minimum synthetic media or on synthetic media lacking lysine. Plates were incubated at 30°C for 48 hours and colonies on each plate were counted. Mating efficiency was calculated as the number of colonies on minimum synthetic media/number of colonies on synthetic media lacking lysine. Results were normalized according the WT strain. Averages from duplicates and standard errors were calculated. The experiments were repeated at least twice. Data shown in Data S2. (TIF) [file pbio.1002012.s006.tif]

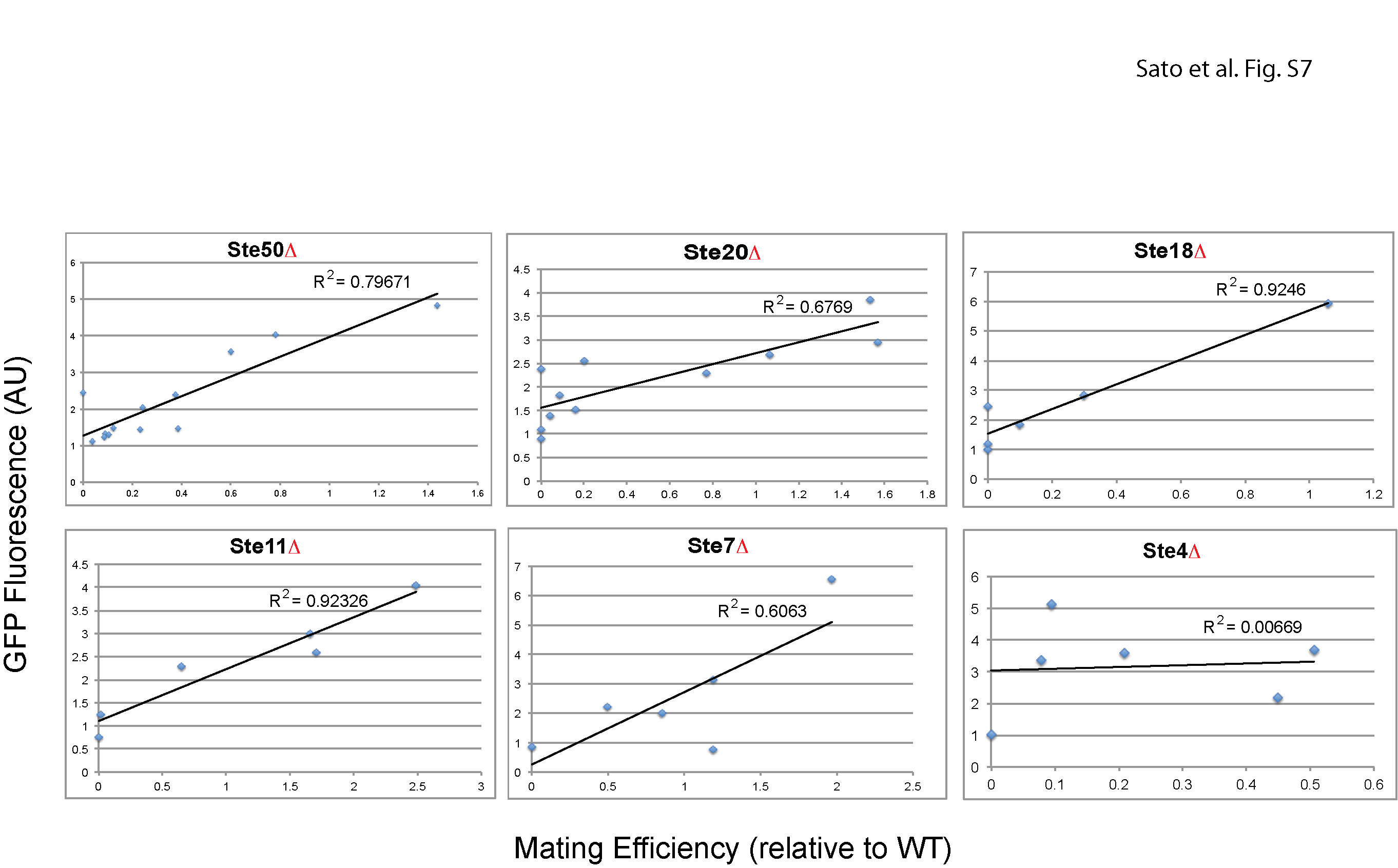

Supplement: Figure S7 — Pathway activation, as determined by GFP fluorescence measured by flow cytometry 2 h after addition of 1 µM pheromone correlates, in most cases, with mating efficiency as determined in quantitative mating assays. Data shown in Data S1 and Data S2. (TIF) [file pbio.1002012.s007.tif]

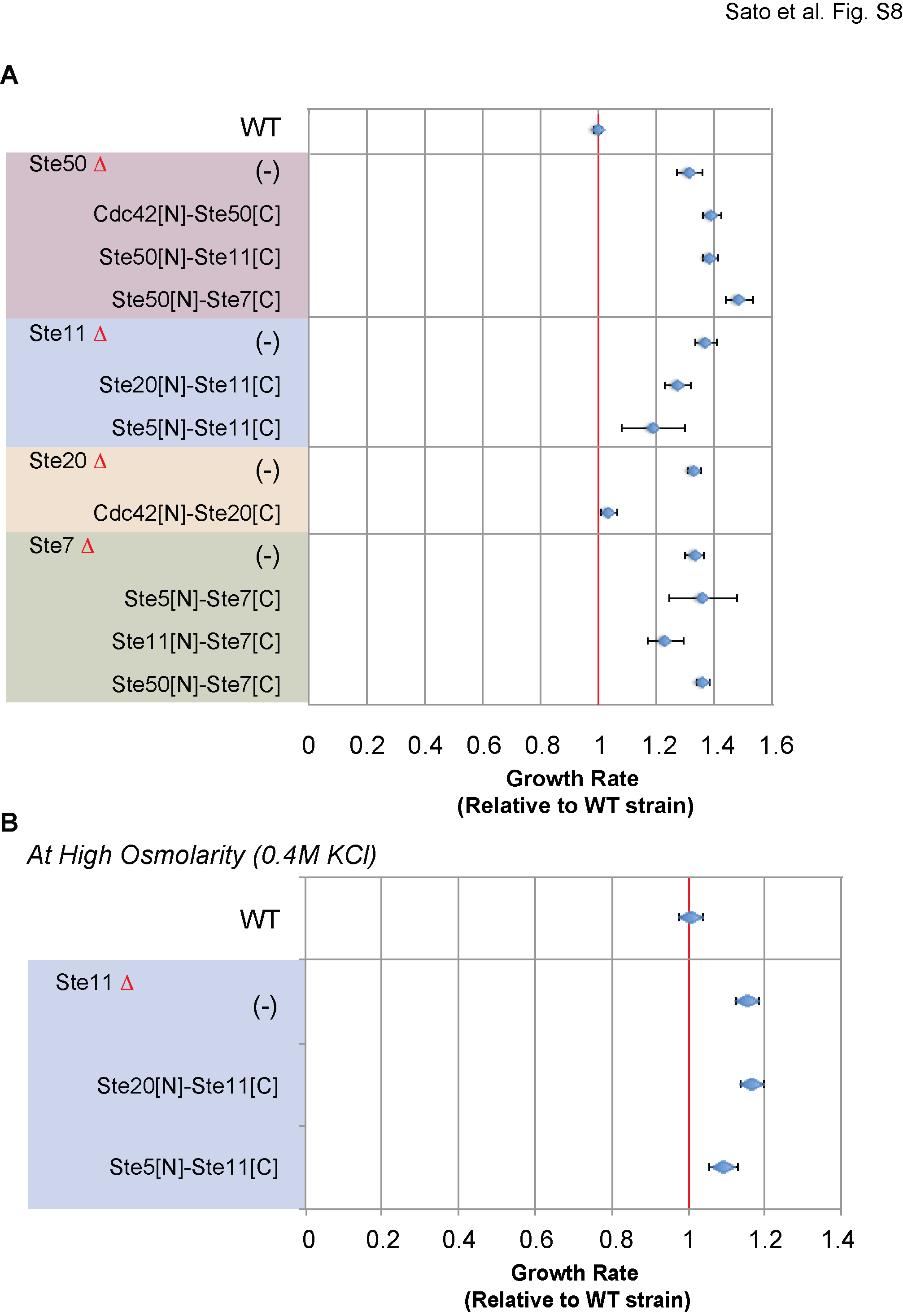

Supplement: Figure S8 — Determination of the growth rates for active domain rearrangement variants, as compared to the growth rates of the WT and the corresponding deletion strains. Each strain was grown in liquid culture (in triplicates) and ODs were measured at 600 nm every hour for 8 hours. Data was fitted using the exponential equation: OD = ODo eλt, where ODo is the initial OD value, λ is the growth rate, and t is time. (A) Cultures were grown in rich media under isosmotic conditions. (B) Cultures were grown in rich media under high osmolarity stress (0.4 M KCl). Data shown in Data S2. (TIF) [file pbio.1002012.s008.tif]

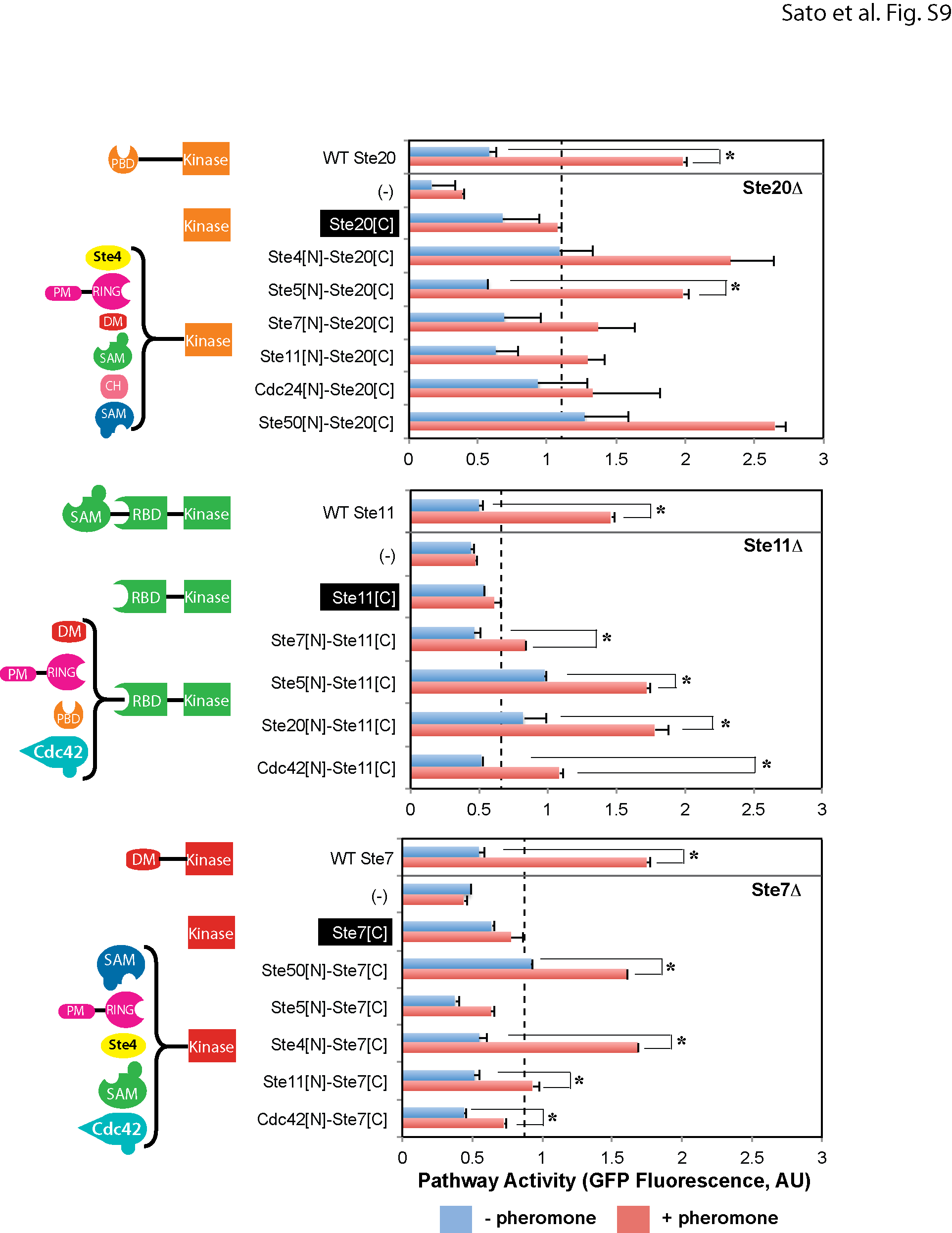

Supplement: Figure S9 — Statistical analysis comparing the GFP values for the variants analyzed in Figure 3A , before or after addition of pheromone. In all cases, two tailed t-tests were performed. Significant differences (p<0.05) are marked with asterisks. Data shown in Data S1 and Data S2. (TIF) [file pbio.1002012.s009.tif]

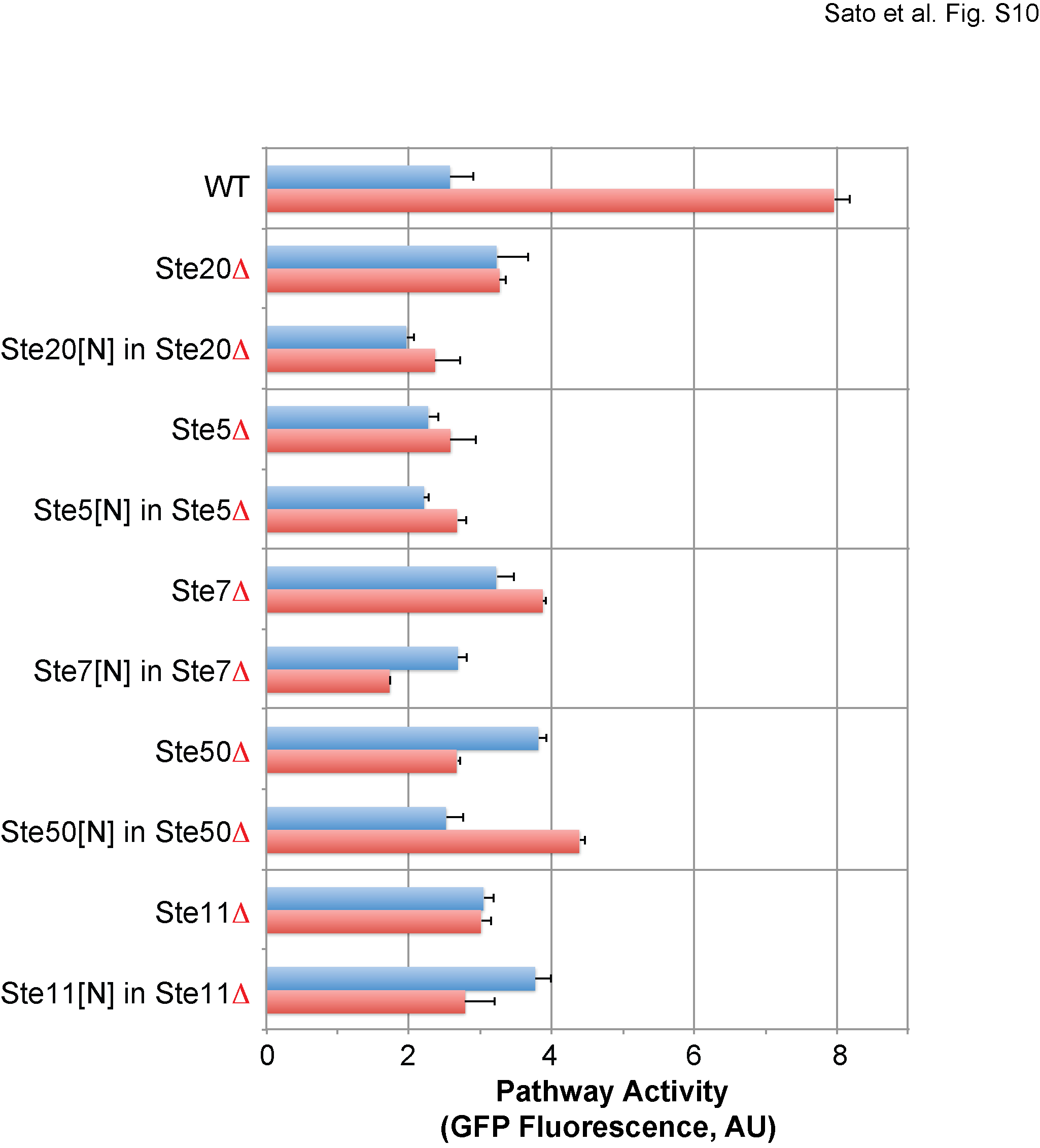

Supplement: Figure S10 — Ability of isolated N-t domains to mediate mating pathway activation. The corresponding deletion strains were transformed with N-t localization domains alone and the fluorescence of the mating pathway reporter pFus1-GFP was measure before or 2 h after addition of pheromone. As shown in the figure, none of the localization domains can restore pathway activity in the deletion strains. Data shown in Data S2. (TIF) [file pbio.1002012.s010.tif]

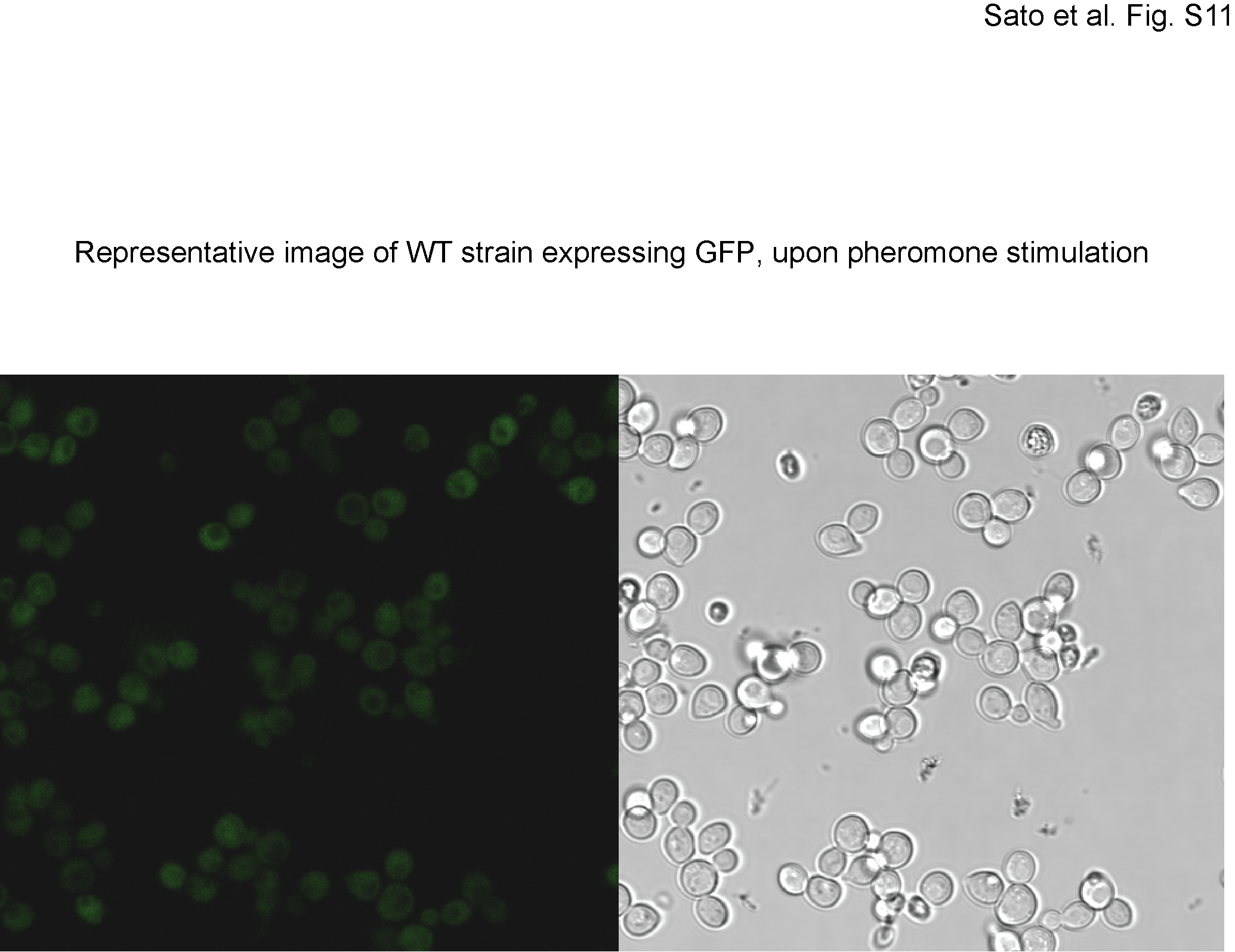

Supplement: Figure S11 — The recruitment of the GFP-tagged variants to the mating projections seen in Figure 3D , depend on the presence of localization domains in the domain rearrangement variants. When GFP is not fused to localization domains, it remains uniformly distributed in the cytoplasm, failing to localize to the mating shmoos. (TIF) [file pbio.1002012.s011.tif]

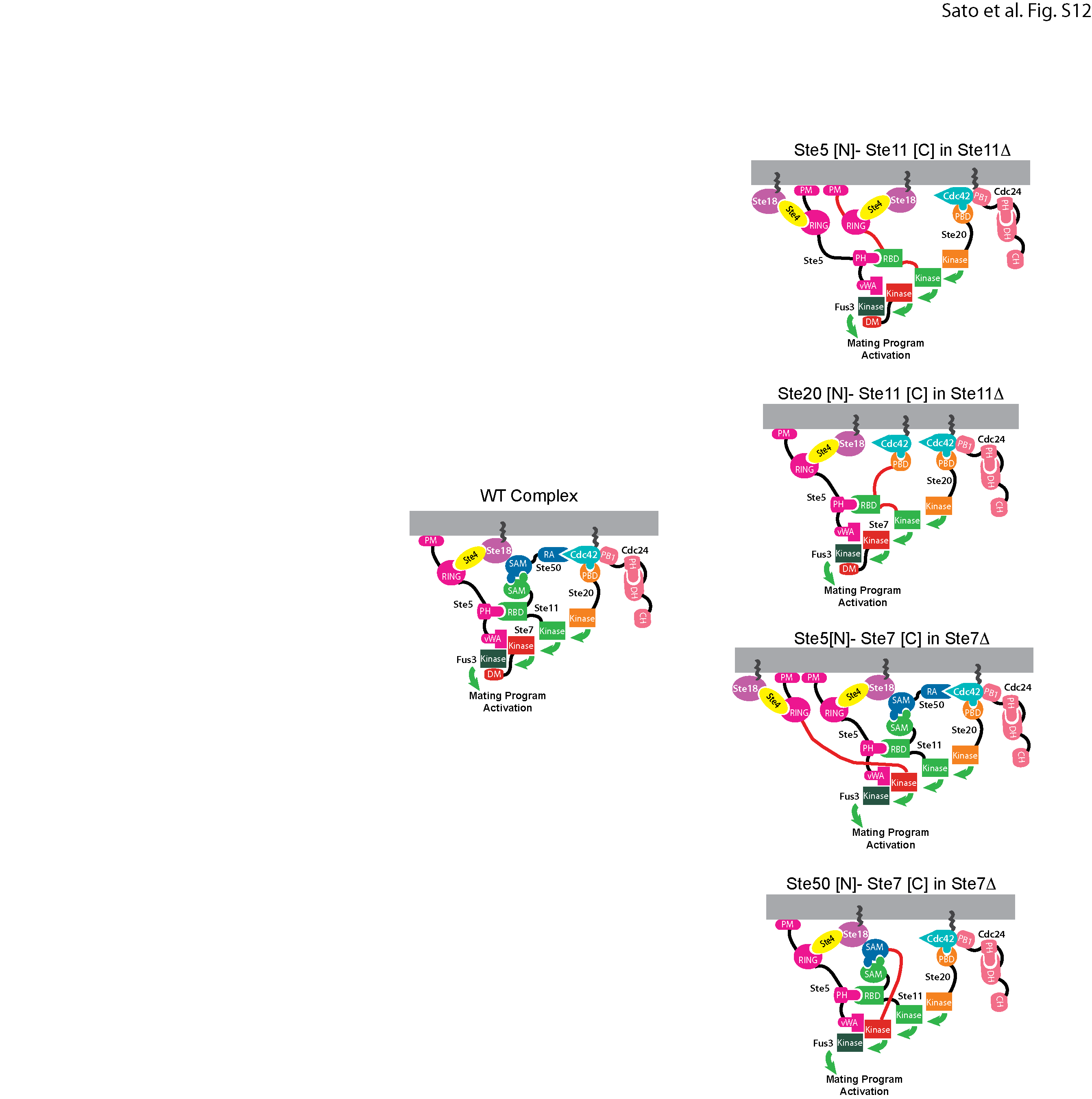

Supplement: Figure S12 — Alternative mechanisms of kinase recruitment to the signaling complex, for the domain shuffling variants shown to localize to the mating projections (shmoos) by fluorescence microscopy. Note that our results suggest that the complex stoichiometry is flexible and can accommodate a diverse number of components. (TIF) [file pbio.1002012.s012.tif]

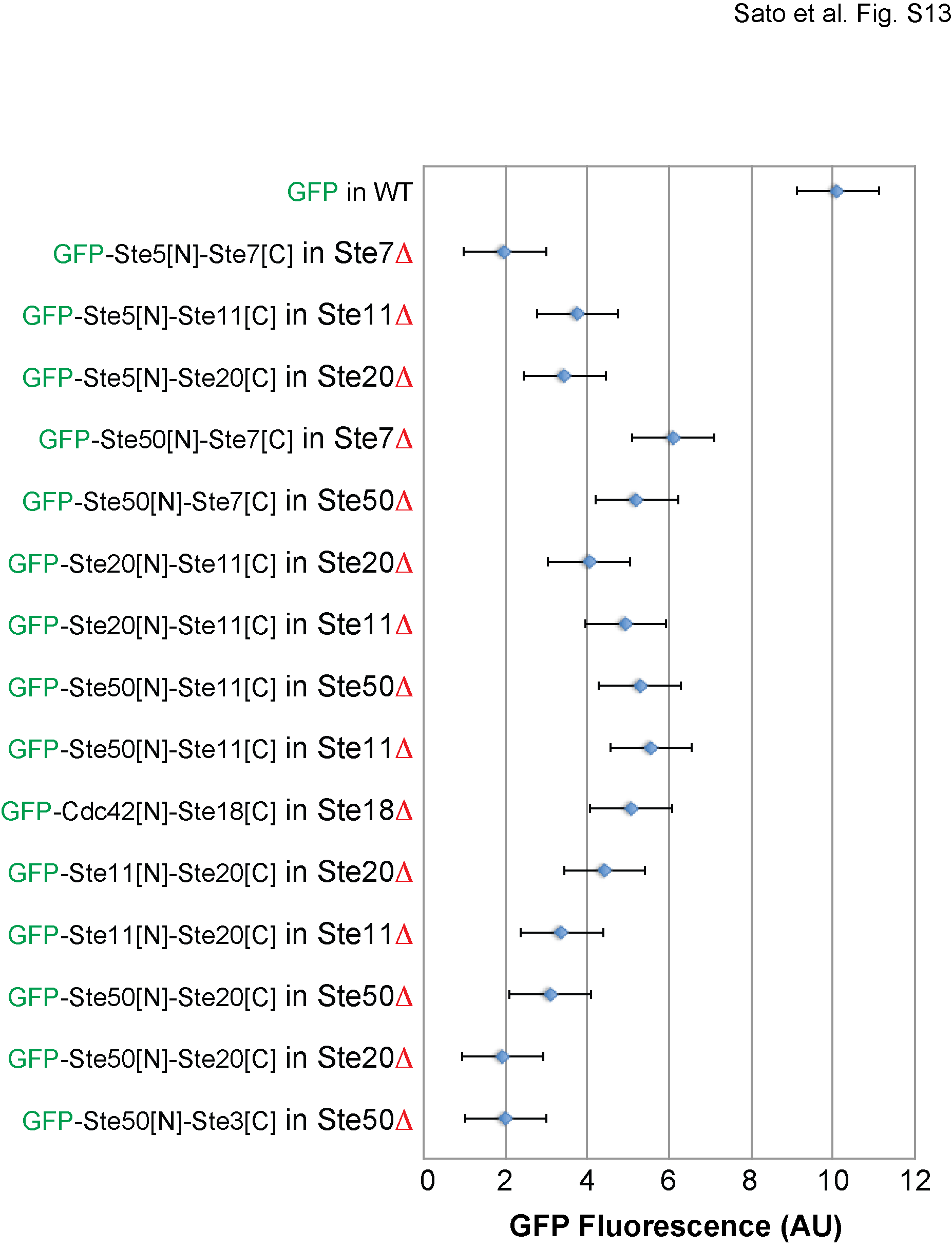

Supplement: Figure S13 — There is no simple correlation between a variant's expression levels and its ability to mediate mating pathway response. Domain rearrangement variants were tagged at their N-t with GFP and transformed in the corresponding deletion strain (note that these strains did not have a mating reporter pFus1-GFP and therefore the only GFP signal measured was derived from the tagged variants themselves). GFP fluorescence was measured by flow cytometry. Data shown in Data S2. (TIF) [file pbio.1002012.s013.tif]

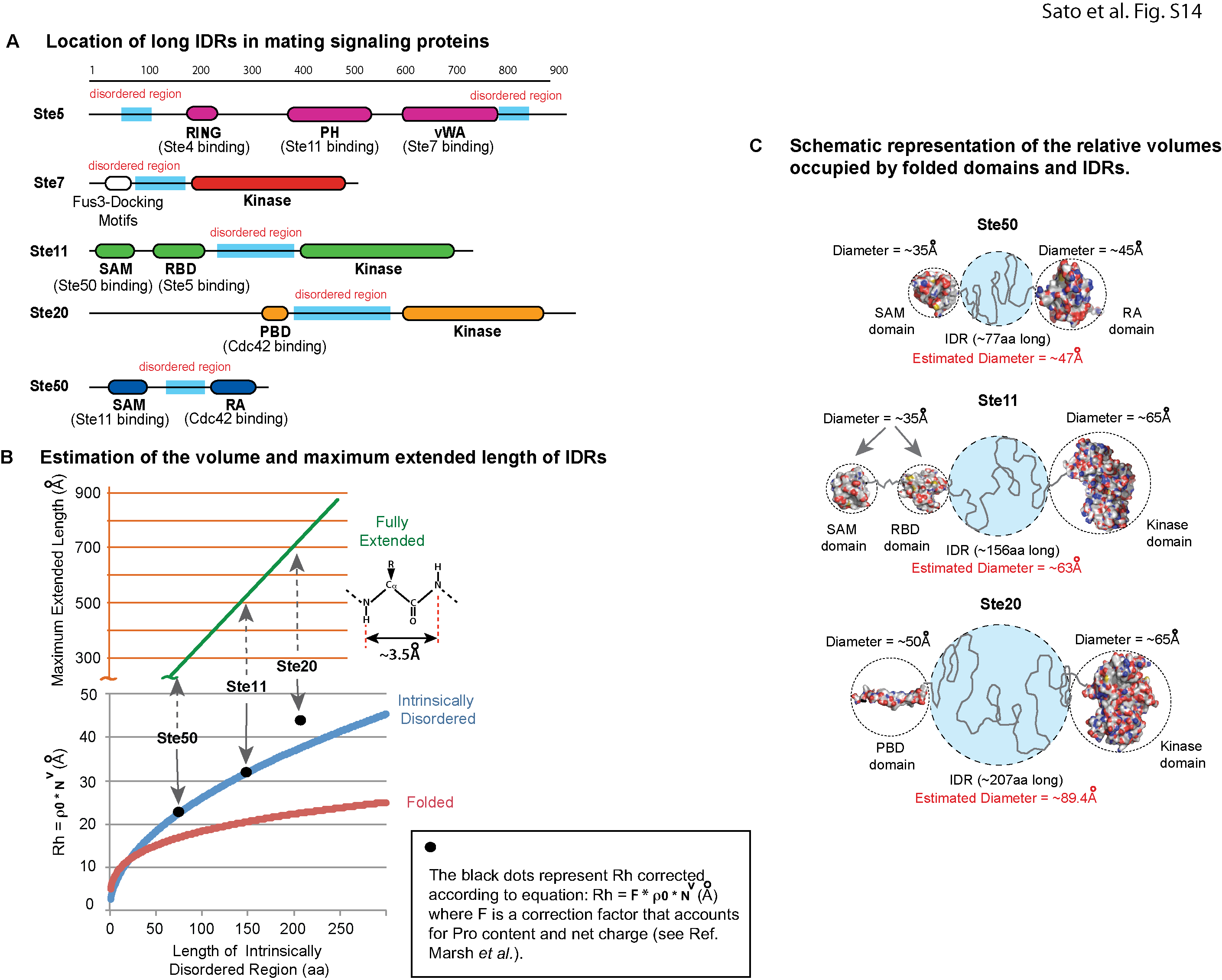

Supplement: Figure S14 — IDRs are found in Ste5, Ste7, Ste11, Ste20, and Ste50. While in Ste7 and Ste5 IDRs are located at the protein termini, in Ste11, Ste20, and Ste50, IDRs are found within inter-domain linkers and, thus, they can separate folded domains by long and flexible distances. (A) Schematic representation of the IDR-containing mating pathway proteins Ste5, Ste7, Ste11, Ste20, and Ste50. IDRs are represented as light blue segments. (B) Estimation of the volume and maximum extended length of Ste11, Ste20, and Ste50 IDRs. (C) Schematic representation of the relative volume occupied by either IDRs or folded domains in Ste11, Ste20, and Ste50. (TIF) [file pbio.1002012.s014.tif]

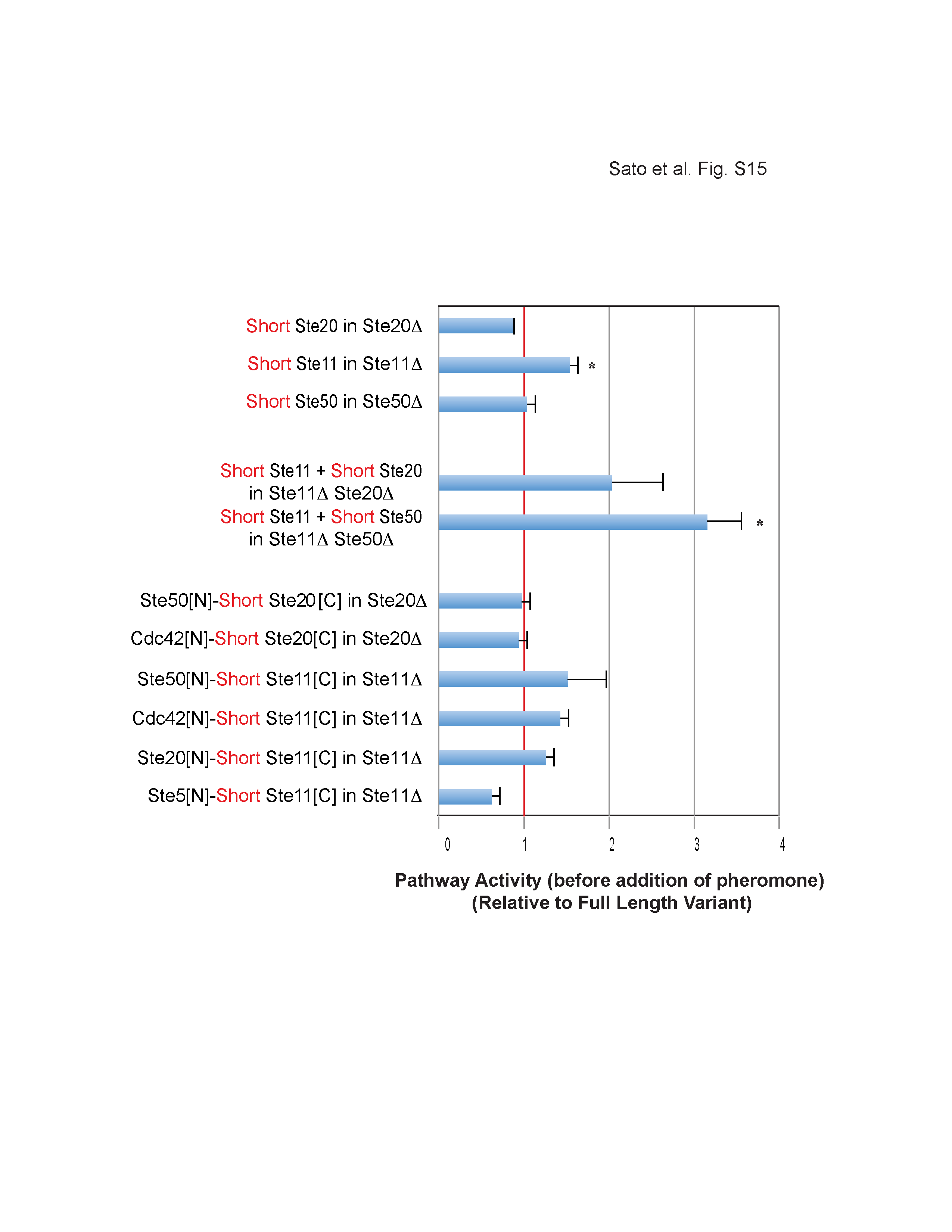

Supplement: Figure S15 — Pathway activation (as determined by GFP fluorescence) for IDR-deleted variants, before the addition of pheromone, as compared with the corresponding full length variants. In the single Δ strains, deletion of Ste20's or Ste50's IDRs does not affect basal levels of pathway activation. In contrast, deletion of Ste11's IDR causes small increases in basal pathway activation, suggesting it may alter the regulation of Ste11's kinase activity. Furthermore, in the double Δ strains, simultaneous deletion of Ste11 and Ste20 IDRs or of Ste11 and Ste50 IDRs leads to large increases in basal levels of pathway activation, suggesting that the simultaneous deletions have a marked effect in the regulation of pathway function. Statistically significant differences are marked with asterisks. Data shown in Data S1. (TIF) [file pbio.1002012.s015.tif]

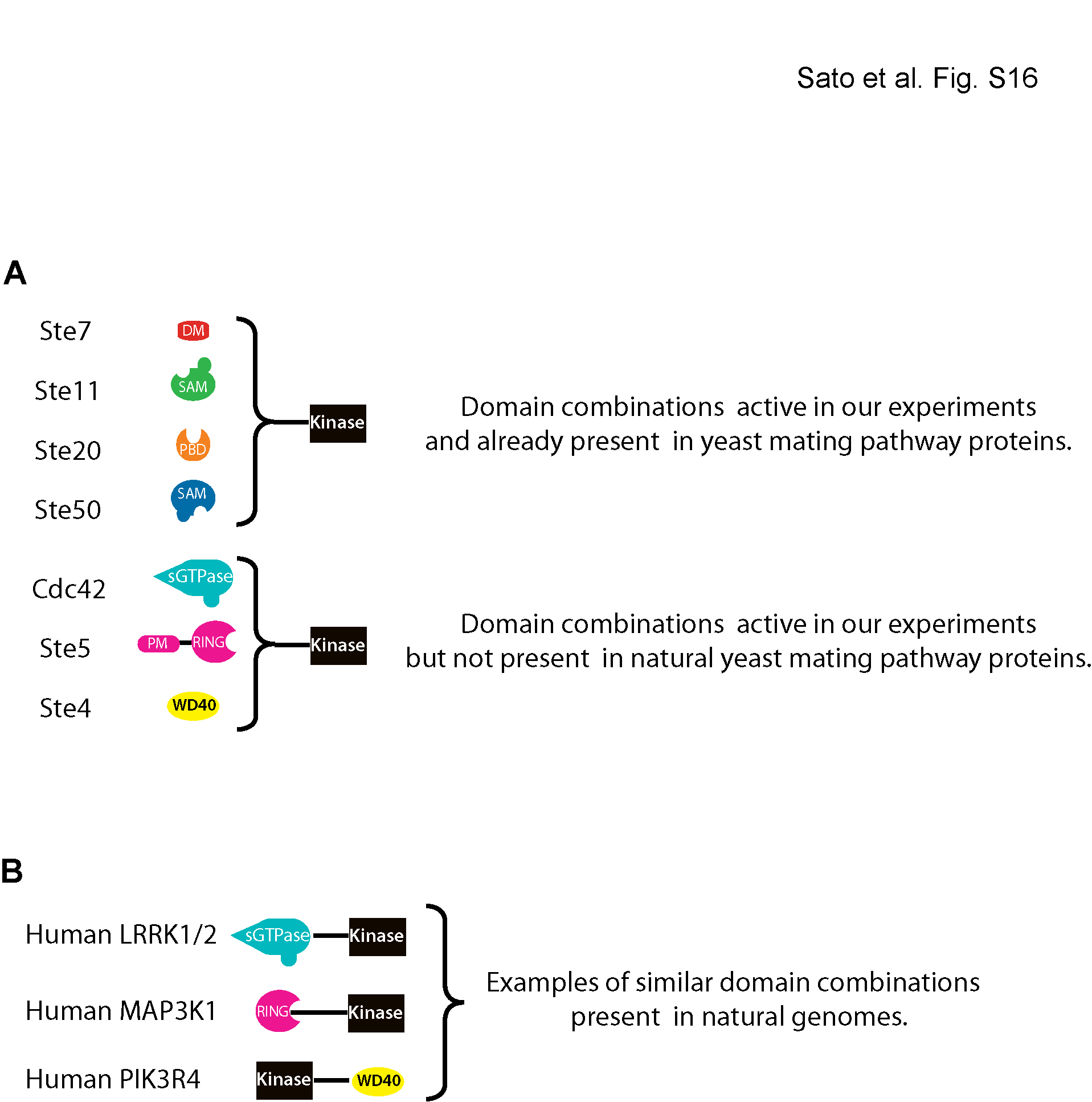

Supplement: Figure S16 — Schematic representation of proteins with alternative domain combinations that lead to functional pathways in our experiments, and are also found in natural proteins. (TIF) [file pbio.1002012.s016.tif]
