# Supplementary material for: The Robustness of a Signaling Complex to Domain Rearrangements Facilitates Network Evolution
Source: PLoS Biol. 2014 Dec 9;12(12):e1002012. doi: 10.1371/journal.pbio.1002012 (PMC4260825; doi:10.1371/journal.pbio.1002012)
Supplement: Text S1 — Supplemental material and methods. (DOCX) [file pbio.1002012.s020.docx]

**SUPPLEMENTAL MATERIALS AND METHODS FOR THE MANUSCRIPT:**

**The Robustness of a Signaling Complex to Domain Rearrangements Facilitates Network Evolution**

Paloma M. Sato, Kogulan Yoganathan, Jae H. Jung and Sergio G. Peisajovich

***Yeast strains***

Deletion strains were derived from a W303 strain with the following genotype: *MATa, bar1::NatR, far1Δ, mfa2::pFUS1-GFP, his3, trp1, leu2, ura3*, for strains to be used in flow cytometry (as the presence of far1 would lead to schmooing which will affect scattering in flow cytometry experiments, and bar1 will alter pheromone concentrations); or from a *trp1, leu2, ura3, his3, ADE2 can1*, for strains to be used in microscopy*.* Seven strains were created in which the following genes from the mating pathway were deleted individually: Ste4, Ste5, Ste7, Ste11, Ste18, Ste20 and Ste50. Individual deletions were done by homologous recombination using Trp as a selectable marker. Deletion strains were validated by genomic PCR and flow cytometry (each individual deletion of a “Ste” gene impaired pathway-dependent GFP expression). Double deletion strains (Ste20Δ Ste11Δ, Ste7Δ Ste11Δ, and Ste50Δ Ste11Δ) were also made by homologous recombination, using Leu as the second selectable marker.

***Domain-rearrangement Libraries***

The domain-rearrangement libraries were designed and constructed using a previously described combinatorial cloning strategy [1]. The sequences of all protein domains used to construct the libraries were deposited in Genbank (see Supplementary Data Set 3 for a complete list of accession numbers). All variants were expressed from centromeric plasmids with Leu selection, under control of a constitutive low expression promoter consisting of a 250bp fragment of the *CycI* promoter, and an AdhI transcription terminator.

***Flow Cytometry***

Each strain carrying an individual deletion (or a double deletion, as in Fig. 5) was transformed with a domain-shuffling variant (or a combination of two, as in Fig. 5) that effectively replaced the deleted gene(s). Starters were grown in duplicates overnight at 30^o^C, 250 RPM, and then diluted 30x in 1 mL of appropriate dropout media. After two hours, cultures were treated with 1 µM α-factor (Zymo Research), while controls were left untreated. Cultures were grown for two more hours, upon which protein synthesis was stopped by addition of cyclohexamide. Cultures were then dispensed into 96-well plates and GFP fluorescence was measured by flow cytometry, using a Miltenyi MACSQuant® VYB instrument (10,000 cells counted for each reading). The GFP signal was averaged for all duplicates and standard errors were calculated. All experiments were repeated at least twice (total n ≥ 4) and found to be in good agreement.

***Fluorescence Microscopy***

All domain-shuffling variants were tagged with GFP at their N-termini, as previously described [1]. Starters were grown overnight, then diluted 100x and grown for 2 more hours, all at 30^o^C, 250 RPM. Cultures were then sonicated briefly to disrupt cell aggregates (2 pulses in a Branson Sonifier 250 with a 2 mm tip, output control 1 and duty cycle 10%). 100 μL of cell cultures were dispensed in individual wells of a concavalin-A coated 96-well plate, and spun down at 1500 RPM for 1 minute to bind cells to the bottom of the plate. Cells were washed twice with fresh media and covered with 100 μL of fresh media with or without 1 μM α-factor, and incubated at 30^o^C for 1-2 hours. Imaging was performed with an automated inverted Leica TCS SP8 confocal microscope using 63x HC PL Apo oil CS2 objective.

***Quantitative Mating Assays***

Mating assays were performed with minor modifications to a previously described method [1]. Specifically, each “a-type” individual deletion strain (SO992, W303-derived, *trp1, leu2, ura3, his3, ADE2 can1*) described above was transformed with appropriate plasmids encoding each domain-rearrangement variant to be tested. Cells were grown overnight at 30°C, 250 RPM, then diluted 10x and grown until OD600 = 0.8 – 1.0. Equal amounts (10^6^) of “A-type” cells transformed with each variant (or controls) were mixed with WT “α-type” cells in a final volume of 200 μl. A quarter of the mixed cells were then deposited on the surface of a polycarbonate filter placed on an YPD plate. The plates with the filters were incubated for 3 hours at 30°C. Cells were then detached from the filters by vortexing for 2 minutes in 3 mL of minimum synthetic media. 250 μL of this solution was then plated on minimum synthetic media and 250 μL on synthetic media lacking lysine. Plates were incubated at 30^o^C for 48 hours and colonies on each plate were counted. Mating efficiency was calculated as the number of colonies on minimum synthetic media divided by number of colonies on synthetic media lacking lysine [2]. Results were normalized according the wild type strain. Averages from triplicates and standard errors were calculated. The experiments were repeated at least twice (n≥6) and found to be in good agreement.

***Site-directed mutagenesis***

Site-direct mutagenesis was done by Quick Change, following the manufacturer’s protocol (Quick Change^®^ II Site-Directed Mutagenesis Kit – Agilent). Briefly, 50 μL PCR reactions were set up using 50 ng of plasmid as template, 125 ng of each primer, 1μL dNTP mix, and 2.5 U of Pfu Ultra HF DNA polymerase. The cycling parameters were: 95^o^C-30 seconds; 16 times of 95^o^C-30 seconds, 55^o^C-1 minute, 68^o^C-10 minutes. The reaction was then treated with DpnI to remove plasmid template. 5 μL of PCR reaction were then transformed in XL1 Blue competent cells. Mutations were verified by DNA sequencing.

***Determination of Growth Rates***

Strains were grown in liquid culture (in triplicates) and ODs were measured at 600nm every hour for 8 hours. Data were fitted using the exponential equation:

OD = OD_o_ e ^λt^  where OD_o_ is the initial OD value, λ is the growth rate, and t is time.

***Identification of Proteins in Natural Genomes with Domain Compositions Similar to Those Found in our Library Screening***

The three novel domain compositions that resulted in active variants in our library correspond to kinase domains combined with a small GTPase domain, a RING domain, or WD-40 repeat-containing β-propeller domain. In order to identify natural proteins with similar domain compositions, we searched the Domain Club Browser Database [3] (<http://pawsonlab.mshri.on.ca/DomainClub/domainClub.php>) for all known domain compositions that included a small GTPase, RING or β-propeller domain, and then among those identified, we searched for those that included a kinase domain as well.

***Estimation of the Volume Occupied by Intrinsically Disordered Regions***

Hydrodynamic Radii for IDRs was calculated using the power law relation Rh = F * ρ_0_ * N^ν^ [4], where ρ_0_ is a constant that depends on persistence length (monomer’s bonds geometry, determined to be 4.92 for folded proteins and 2.49 for IDR), N is the number of residues in the polymer, ν is a scaling factor determined experimentally to be ~0.285 for folded proteins and ~0.509 for IDR, and F is a correction factor that accounts for the net charge and Pro content of the IDR (F = (A * P pro + B) (C * Q + D), where P pro is the fraction of proline residues in the IDR, Q is the absolute value of the net charge within the IDR, and A, B, C and D are fitting constants [4]).

***Deletion of Intrinsically Disordered Regions***

The intrinsically disordered regions (IDRs) of Ste20 and Ste11 were identified in the Pfam database (<http://pfam.sanger.ac.uk/protein/P23561>). Specifically, we deleted the disordered regions between amino acids 408 and 578 in Ste20, and between 258 and 354 in Ste11, in both cases by inserting (using site directed mutagenesis) two PmeI restrictions sites in frame, followed by digestion with PmeI and self-ligation. All shortened variants were verified by DNA sequencing.

**SUPPLEMENTAL REFERENCES**

1. Peisajovich, S.G., Garbarino, J.E., Wei, P., and Lim, W.A. (2010). Rapid diversification of cell signaling phenotypes by modular domain recombination. Science *328*, 368-372.

2. Sprague, G.F., Jr. (1991). Assay of yeast mating reaction. Methods in Enzymology *194*, 77-93.

3. Jin, J., Xie, X., Chen, C., Park, J.G., Stark, C., James, D.A., Olhovsky, M., Linding, R., Mao, Y., and Pawson, T. (2009). Eukaryotic protein domains as functional units of cellular evolution. Science Signaling *2*, ra76

4. Marsh, J.A., and Forman-Kay, J.D. (2010). Sequence determinants of compaction in intrinsically disordered proteins. Biophysical journal *98*, 2383-2390.
